# Florfenicol-Polyarginine Conjugates Exhibit Promising Antibacterial Activity Against Resistant Strains

**DOI:** 10.3389/fchem.2022.921091

**Published:** 2022-07-01

**Authors:** Zhun Li, Ya-Jun Yang, Zhe Qin, Shi-Hong Li, Li-Xia Bai, Jian-Yong Li, Xi-Wang Liu

**Affiliations:** Key Lab of New Animal Drug Project of Gansu Province, Key Lab of Veterinary Pharmaceutical Development of Ministry of Agriculture and Rural Affairs, Lanzhou Institute of Husbandry and Pharmaceutical Science of Chinese Academy of Agricultural Sciences, Lanzhou, China

**Keywords:** florfenicol, polyarginine, conjugates, resistant strains, prodrugs

## Abstract

Florfenicol was widely used as antibiotic in the livestock and poultry breeding industry, resulting in a serious problem of drug resistance. In order to solve the resistance of florfenicol, this study designed and synthesized a new series of florfenicol-polyarginine conjugates and tested for antimicrobial activities. Drug-sensitive bacteria, gram-negative bacteria *Escherichia coli* (*E. coli*) and gram-positive *Staphylococcus aureus* (*S. aureus*), were sensitive to several of the compounds tested. These conjugates also showed excellent activity against drug-resistant strains such as methicillin-resistant *S. aureus* (MRSA) and florfenicol resistant *Escherichia coli* strains (2017XJ30, 2019XJ20), one of which as E6 had a minimum inhibitory concentration of 12.5 μmol/L. These conjugates did not allow bacteria to develop resistance and also decreased bacterial growth by membrane depolarization and disruption. Additionally, florfenicol succinate (C1) showed certain activity after coupling with arginine. This suggested that conjugating arginine to florfenicol succinate effectively modulated the properties of prodrugs. These new conjugates may provide useful insights for expanding the pool of antibiotics.

## Introduction

The drug resistance of bacteria to antibiotics is becoming more and more serious, which poses a threatening for human and animals (Martens and Demain, 2017; Aslam et al., 2018). The issue has been identified as a major concern by multiple organizations and governments, who have implemented “global action plans” and various initiatives to combat and ameliorate the rise of antimicrobial resistance (Mendelson and Matsoso, 2015; Organization for Economic Cooperation and Development (OECD), 2018; Centers for Disease Control and Prevention, 2020). There exists an urgent need for the development of novel antimicrobials to treat drug-resistant bacterial infections (World Health Organization, 2017; WHO, 2020; Tague et al., 2021).

Florfenicol is widely used as antibiotic in the livestock and poultry breeding industry because of its broad antibacterial spectrum and good efficacy, resulting in a serious problem of drug resistance (Du et al., 2004; Singer et al., 2004; Liu et al., 2013). Owing to very low water solubility, the development of water-soluble florfenicol with excellent performance has always been a research hotspot in the field of veterinary medicine (Schwarz et al., 2004; Glinka and Zhang, 2011; Zhang et al., 2020). Antimicrobial peptides (AMPs) exist in most organisms and have broad-spectrum antibacterial effect, which can resist the invasion of external bacteria, fungi, protozoa and viruses (Carratalá et al., 2020; Chu et al., 2020; da Silva et al., 2020; de Azevedo dos Santos et al., 2020; Thapa et al., 2020). Because the target of AMPs is bacterial cell membrane, which is different from the antibacterial mechanism of traditional antibiotics, they are expected to become the most promising new antibiotics in the post antibiotic era (Brogden, 2005; Mahlapuu et al., 2020; Li et al., 2021; Liu et al., 2021). AMPs primarily operated through membrane destabilization and disruption mechanisms, and different AMPs had the different effects on membrane integrity and phospholipid membrane interactions (Lin et al., 2022). Cell-penetrating peptides (CPPs) are commonly small cationic peptides that interact with membrane and frequently have amphiphilic properties (Ramsey et al., 2015). Polyarginine peptides are widely used as CPPs and shown to inhibit bacteria by entering certain microbial cell membranes (Guzmán et al., 2013; Sparr et al., 2013), for example, polydopamine-decorated nanoparticles have exhibited a strong antimicrobial activity against *S. aureus* (Muller et al., 2020). The key to the initial interaction between AMPs and bacterial membrane is the high content of cationic charges, and the hydrophobic moieties drive peptides into bacterial membrane, resulting in membrane permeabilization (Michael, 2002; Wimley, 2010). Therefore, conjugation of hydrophobic moieties with polyarginine peptides may form amphiphilic hybrid molecules, which can improve the interaction with membrane and antibacterial activity.

Peptide-drug conjugate (PDC) is a new class of drug molecules that connect small molecule drugs with polypeptides through chemical bonds (Ma et al., 2017; Wang et al., 2017). PDC relies on the self-properties of peptides to effectively improve the shortcomings of small molecule drugs, such as enhancing the water solubility and targetability of drugs (Craik and Fairlie, 2013; Cooper et al., 2021). Vancomycin−D-octa arginine conjugate was a dual-function conjugate and showed greater cellular accumulation and membrane perturbation compared to vancomycin (Antonoplis et al., 2018). Chen et al. designed and synthesized a bacteria-targeting conjugate, based on AMPs for bacteria diagnosis and therapy, which reduced the toxicity and enhanced the antibacterial activity of chloramphenicol (Chen et al., 2015). A series of novel florfenicol-polyarginine conjugates were synthesized and designed in this study, and their antibacterial, mechanism of action, and cytotoxicity to Caco-2 cells were evaluated.

## Results and Discussion

### Synthesis and Characterization

**SCHEME 1:**
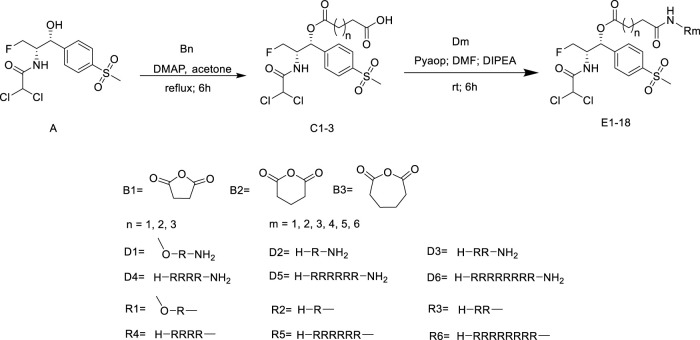
Synthesis of the florfenicol-polyarginine conjugates E1-18.

All of the peptide-florfenicol conjugates were synthesized by esterification (Okuno et al., 2015; Sehaqui et al., 2017) and amidation (Albericio, 2004; Antonoplis et al., 2018) reaction. Firstly, compound C1-3 were formed by esterification of the hydroxyl position of florfenicol with succinic anhydride, glutaric anhydride and hexanedioic anhydride (Okuno et al., 2015). Secondly, Arginine polypeptide modifiers D1-6 were obtained by Fmoc solid phase synthesis (Antonoplis et al., 2018). In the experiment, the arginine residues of the six peptide precursors were 1, 2, 4, 6 and 8, respectively. Lastly, peptide-florfenicol conjugates E1-18 were synthesized by amidation of C1-3 and D1-6 (Albericio, 2004) (Scheme 1). Consequently, 18 peptide-florfenicol conjugates were synthesized. These compounds were purified using preparative reversed-phase High Performance Liquid Chromatography (HPLC), and the purity (>95%) were determined by analytical HPLC. The chemical structures were confirmed by HR-MS, ^1^H NMR and ^13^C NMR. The corresponding chemical structures of conjugates were listed in Table 1.

**TABLE 1:** Chemical structures of conjugates.

Compounds	Chemical structures
E1 (*n* = 1), E7 (*n* = 2), E13 (*n* = 3)	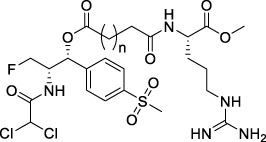
E2 (*n* = 1), E8 (*n* = 2), E14 (*n* = 3)	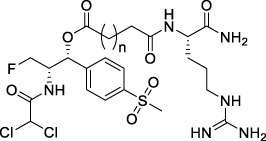
E3 (*n* = 1), E9 (*n* = 2), E15 (*n* = 3)	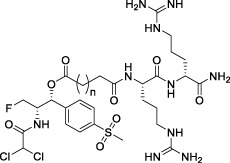
E4 (*n* = 1), E10 (*n* = 2), E16 (*n* = 3)	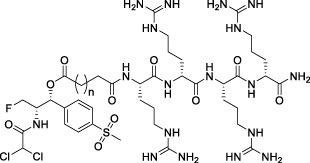
E5 (*n* = 1), E11 (*n* = 2), E17 (*n* = 3)	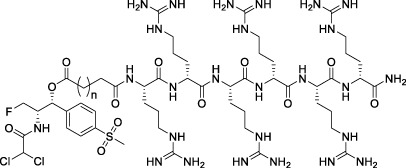
E6 (*n* = 1), E12 (*n* = 2), E18 (*n* = 3)	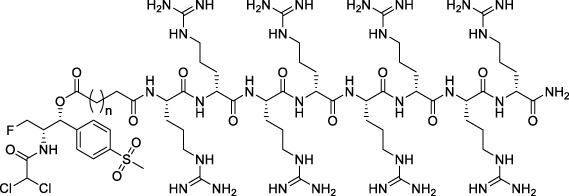

### Antibacterial Activity

All the florfenicol-polyarginine conjugates were tested against Gram-positive and Gram-negative bacteria, including MRSA (ATCC 43300), *S. aureus* (ATCC 25923), *E. coli* (ATCC 25922), clinical strains of *E. coli* (2019XJ06, 2019XJ25, 2018XJ108, 2018XJ105, 2018XJ30) and clinical strains of florfenicol resistant *E. coli* (2017XJ30, 2019XJ20). The antibacterial efficacy of these florfenicol-polyarginine conjugates were determined in Muller-Hinton broth (MHB) culture medium and demonstrated with their minimum inhibitory concentrations (MICs)(Zhang et al., 2018) (Table 2).

**TABLE 2:** Minimal inhibitory concentrations (MICs) of the compounds.

Compound	MIC(µmol/L)									
	*E.coli* (ATCC 25922)	*S. aureus* (ATCC 292130)	MRSA (ATCC 43300)	2017XJ30	2019XJ	2019XJ	2019XJ25	2018XJ108	2018XJ105	2018XJ30
					20	06				
Florfenicol	6.25	6.25	6.25	>100	>100	6.25	6.25	6.25	6.25	6.25
C1	>100	>100	>100	>100	>100	>100	>100	>100	>100	>100
C2	>100	>100	>100	>100	>100	>100	>100	>100	>100	>100
C3	>100	>100	>100	>100	>100	>100	>100	>100	>100	>100
D1	>100	>100	>100	>100	>100	>100	>100	>100	>100	>100
D2	>100	>100	>100	>100	>100	>100	>100	>100	>100	>100
D3	>100	>100	>100	>100	>100	>100	>100	>100	>100	>100
D4	>100	>100	>100	>100	>100	>100	>100	>100	>100	>100
D5	100	50	50	100	100	100	100	100	100	100
D6	50	25	25	50	50	50	50	50	50	50
E1	25	50	50	>100	>100	25	25	25	25	25
E2	25	25	25	>100	>100	25	25	25	25	25
E3	25	25	25	>100	>100	25	25	25	25	25
E4	25	25	25	>100	>100	25	25	25	25	25
E5	25	25	25	100	100	25	25	25	25	25
E6	12.5	12.5	12.5	12.5	12.5	12.5	12.5	12.5	12.5	12.5
E7	>100	>100	>100	>100	>100	>100	>100	>100	>100	>100
E8	>100	>100	>100	>100	>100	>100	>100	>100	>100	>100
E9	>100	>100	>100	>100	>100	>100	>100	>100	>100	>100
E10	>100	>100	>100	>100	>100	>100	>100	>100	>100	>100
E11	25	50	50	50	50	50	50	25	50	50
E12	12.5	12.5	12.5	12.5	12.5	12.5	12.5	12.5	12.5	12.5
E13	>100	>100	>100	>100	>100	>100	>100	>100	>100	>100
E14	>100	>100	>100	>100	>100	>100	>100	>100	>100	>100
E15	>100	>100	>100	>100	>100	>100	>100	>100	>100	>100
E16	100	>100	>100	>100	>100	100	100	100	100	100
E17	50	100	100	50	50	50	50	50	50	50
E18	12.5	25	25	12.5	12.5	12.5	12.5	12.5	12.5	12.5

The compounds C1-3 had no activity against 10 strains tested (MIC > 100 μmol/L). Among the Dm series polyarginine, D5 and D6 had certain activity against the above bacteria (MIC = 25–100 μmol/L), and the other four had no activity or weak activity (MIC > 100 μmol/L). When compounds C1-3 were conjugated with Dm series polypeptides through amidation reaction, the florfenicol-polyarginine conjugates E1-E18 were obtained. Among them, E6, E12 and E18 had good activity against the tested bacteria, and also had good activity against florfenicol resistant *Escherichia coli* strains: 2017XJ30 and 2019XJ20 (MIC = 12.5 μmol/L), the activity of E5, E11 and E17 were weaker than that of E6, E12 and E18. The conjugates E7-10 and E13-16 had no activity or weak activity (MIC > 100 μmol/L) against the above bacteria. Nevertheless, the conjugates E1-4 showed good antibacterial activity against the above bacteria (MIC = 25 μmol/L) except florfenicol resistant *Escherichia coli* strains 2017XJ30 and 2019XJ20 (MIC > 100 μmol/L). The compound C1-3 were the prodrug of florfenicol, and their activity were lost. Inactive C1 showed certain activity after coupling with inactive polyarginine D1-4, C2 and C3 had no similar effect. This indicated that different links had a great influence on the activity, and conjugating arginine to florfenicol succinate effectively modulated the properties of prodrugs.

### Hemolytic Activity

**FIGURE 1:**
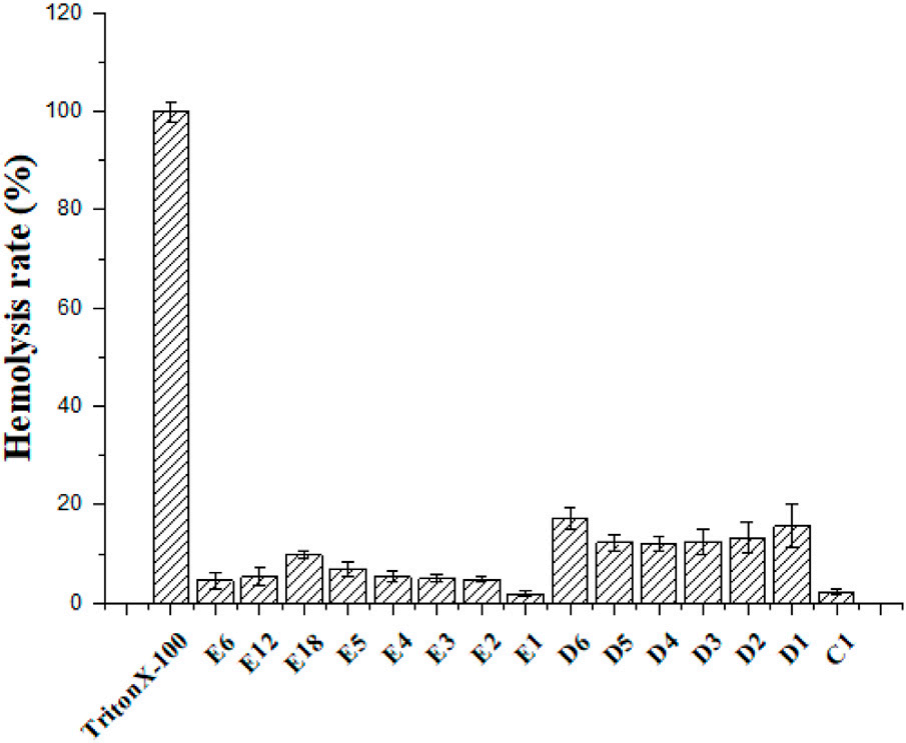
Hemolytic activity of the partial compounds.

Hemolysis was used to evaluate the toxicity of cationic peptides toward mammalian cells (Wimley, 2010). The hemolytic activity of the partial conjugates was represented by their HC_50_ values. The partial conjugates were determined at concentrations ranging from 6.25 to 1000 μmol/L. According to Figure 1, hemolytic concentration (HC_50_) values of conjugates were > 1000 μmol/L. These compounds had not significant effect on mammalian erythrocytes, even at a concentration of 1000 μmol/L. The C1 series conjugates showed lower hemolysis compared with the various numbers of polyarginine peptides. It can be seen from E6, E12 and E18 that the length of link has little effect on hemolysis.

### Cytotoxicity

**FIGURE 2:**
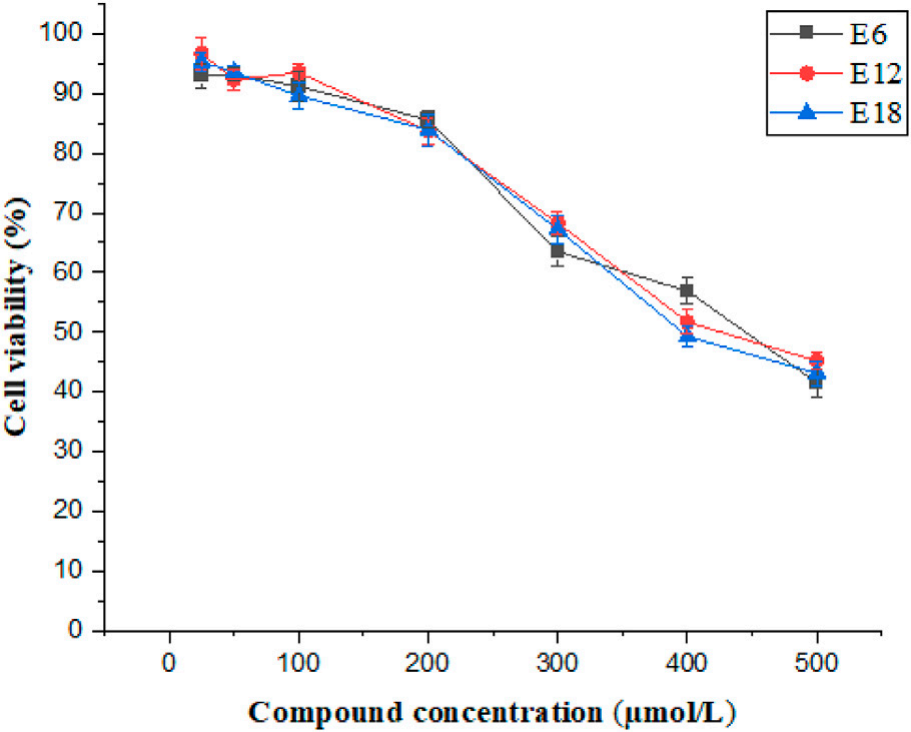
Cell viability of Caco-2 cells after treatment of different concentrations of E6, E12 and E18.

Cytotoxic assays analyzed the selectivity of the compounds toward bacteria and mammalian cells. The conjugates (E6, E12 and E18) were tested on the mammalian cell line Caco-2 by CCK-8 assay and cell viability was more than 50% at 400 μmol/L of E6, E12 and E18 (Figure 2). The results suggested that E6, E12 and E18 exhibited excellent selectivity toward bacterial cells rather than mammalian cells.

### Propensity to Induce Bacterial Resistance

**FIGURE 3:**
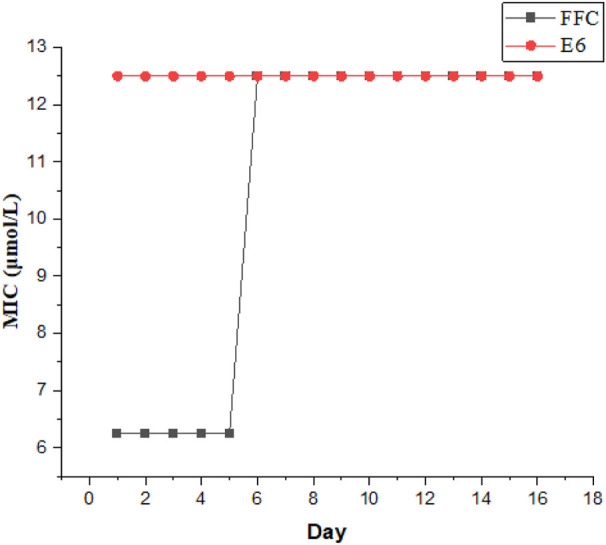
Propensity of *E. coli* resistance against conjugate E6. the control was FFC.

It is crucial to evaluate the potential emergence of resistance from these compounds. We chose E6 as the most active compound to evaluate the ability of the conjugates for suppressing the resistance development against Gram-negative *E. coli*., while florfenicol (FFC) was used as a control antibiotic. As illustrated in Figure 3, after the initial MIC experiment, serial passage was investigated by transferring the growing bacterial suspension at sub-MIC of the compounds (0.5 MIC) and MIC in every passage was determined again. The process was repeated for 16 passages. After 16 passages, the MIC of E6 showed no change against *E. coli*, whereas the MIC of FFC increased by 2-fold. It suggested that these conjugates could be not easy to induce bacterial resistance. However, *E. coli* became resistant to FFC after only a few days. Therefore, these conjugates can be to combat drug-resistant bacteria.

### Plasma Stability

**FIGURE 4:**
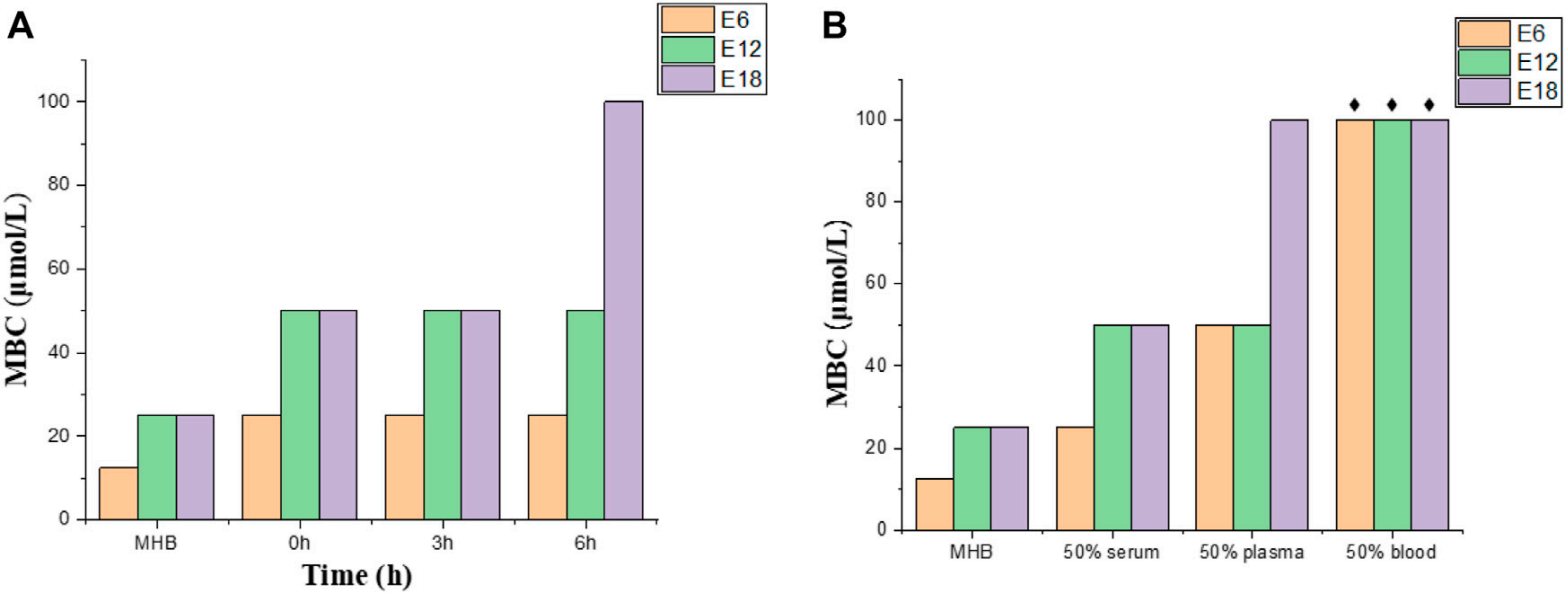
Plasma stability and bactericidal activity in complex mammalian fluids. **(A)** Conjugates (E6, E12, E18) against *E. coli* in plasma treated at different times. **(B)** Conjugates (E6, E12, E18) against *E. coli* in different culture mediums (50% serum, 50% plasma and 50% blood). The black star represents > 100 μmol/L.

Protease degradation is one of the main factors limiting antimicrobial peptides activity in mammalian fluids (Zhang et al., 2018). To determine the plasma stability of conjugates, antibacterial efficacy of the compounds (E6, E12, E18) against *E. coli* were evaluated by preincubating compounds at 37°C for different periods of time (0, 3 and 6 h) in 50% plasma. The MBCs of compounds E6, E12 and E18 increased from 12.5 or 25 μmol/L (100% media) to 50 or 100 μmol/L (50% plasma) after treatment (0, 3 and 6 h) (Figure 4A). Based on the above results, conjugates E6, E12 and E18 lost some antibacterial efficacy upon plasma pretreatment.

### Antibacterial Activity in Complex Mammalian Fluids

Complex mammalian fluids commonly lead to the decrease of antibacterial activity of the peptide. Antibacterial activities of conjugates (E6, E12, E18) were tested by MBC in 50% blood, 50% plasma and 50% serum supplemented with 50% MHB. The MBC values of compounds E6, E12 and E18 increased by 1-fold in serum and 2-fold or 4-fold in plasma, whereas the MBC values increased more than 8-fold in blood (Figure 4B). The combination of conjugates and negative charge macromolecules and proteins may be the reason for the MBC value increase in complex mammalian fluids. Therefore, E6, E12 and E18 were active in serum and plasma, but their antibacterial activities were deprived in blood.

### Bacterial Time-kill Kinetics

**FIGURE 5:**
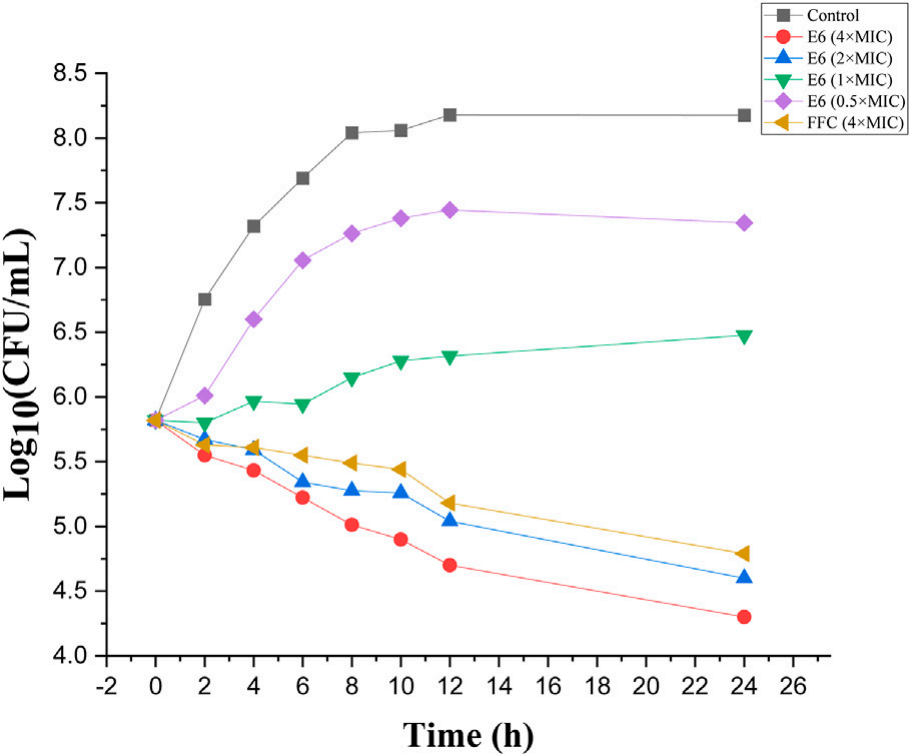
Time-dependent killing by conjugate E6, florfenicol (FFC) and the control against *E. coli*.

Compound E6 displayed promising antibacterial activities and was further evaluated *in vitro* time-kill assay. The negative and positive controls were MHB media and FFC (4 × MIC), respectively. The time-kill curves of different concentrations of E6 against *E. coli* (ATCC 25922) displayed concentration-dependent bacteriostatic effects (Figure 5). Although 0.5 × and 1 × MIC of compound E6 slowed bacterial propagation when compared to negative control, the higher concentrations (2 × and 4 × MIC) showed relatively good bacteriostatic kinetics against *E. coli*. The bacteriostatic effect of E6 (2 × and 4 × MIC) was better than that of FFC (4 × MIC).

### Mechanism of Action

**FIGURE 6:**
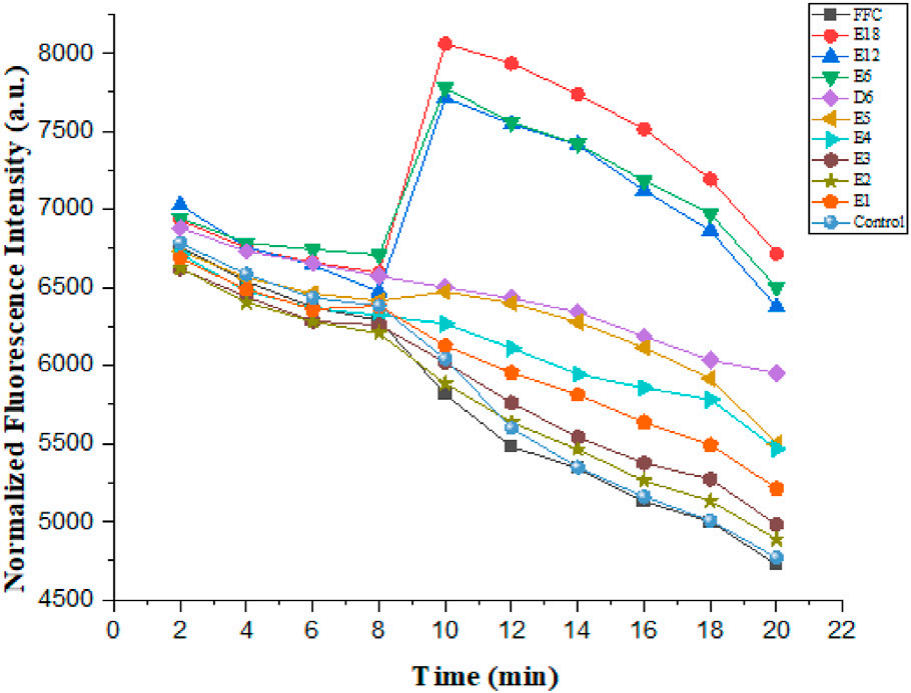
Cytoplasmic membrane depolarization of *E. coli*.

**FIGURE 7:**
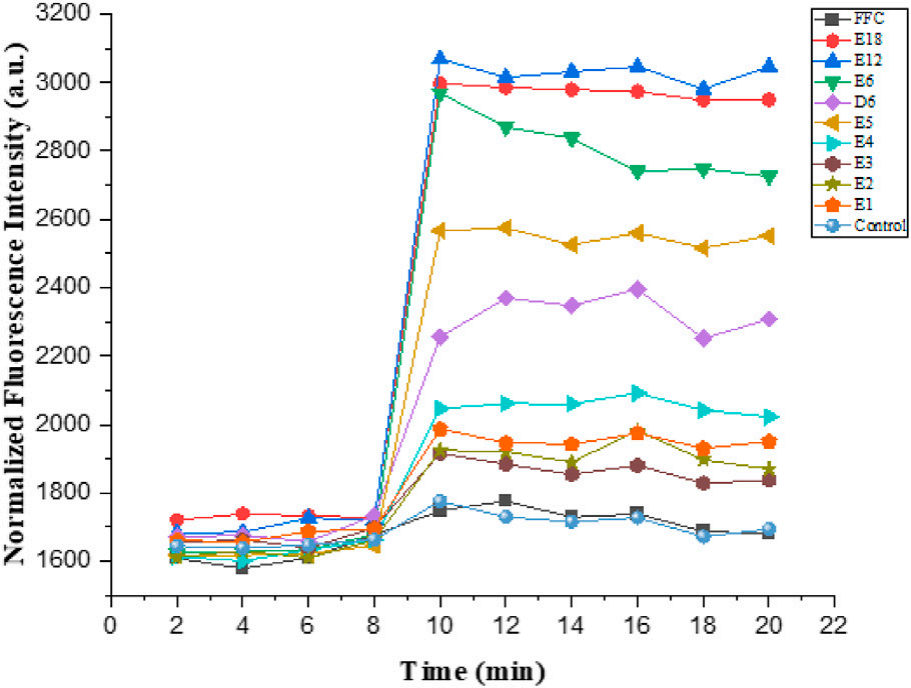
Outer membrane permeabilization of *E. coli*.

**FIGURE 8:**
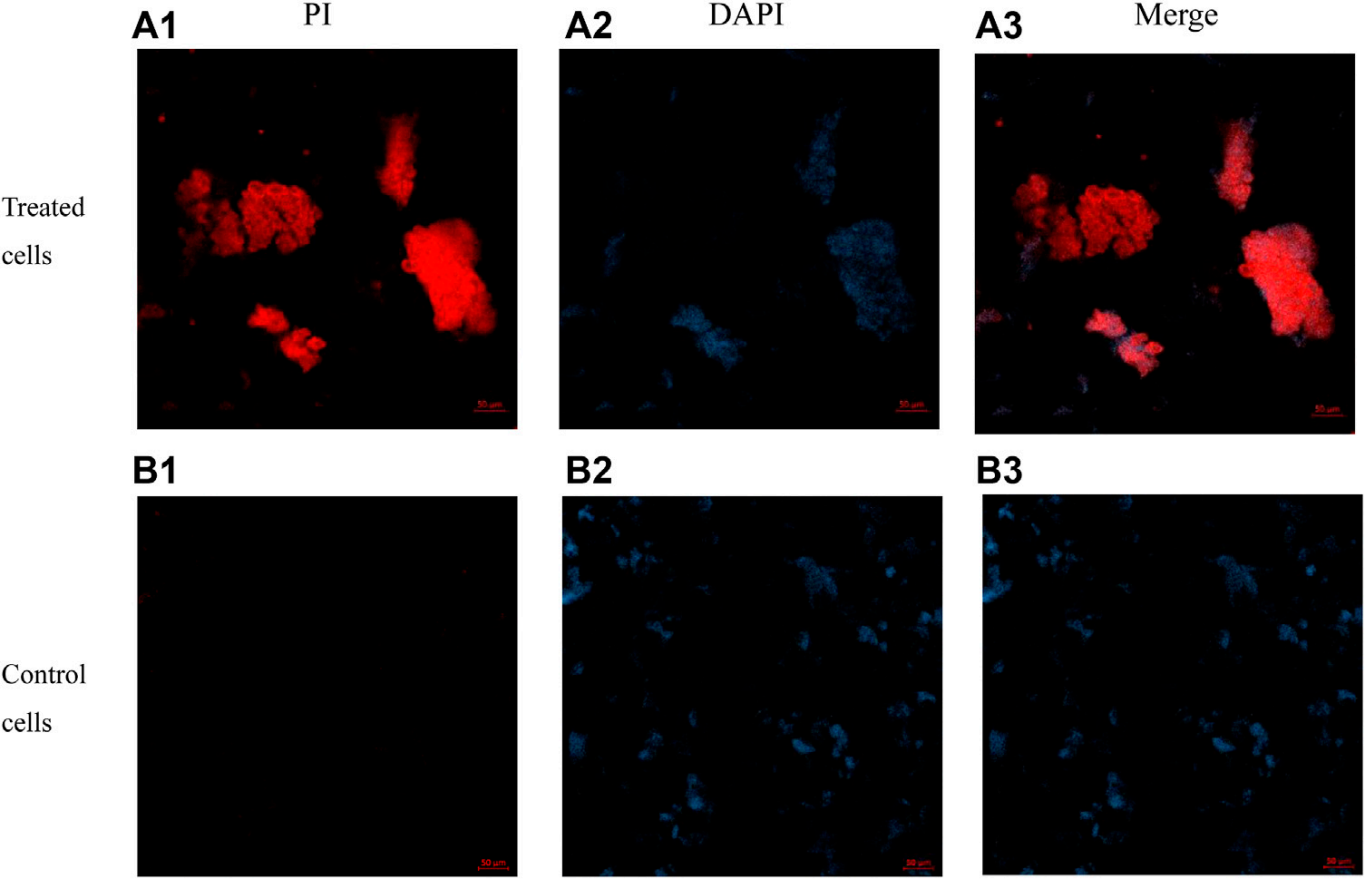
Images of *E. coli* cells treated with 4 × MIC of E6 for 2 h post-incubation acquired by concentric laser scanning microscopy. images (a1–a3) of E6 treated cells stained with PI, DAPI and merged. images (b1–b3) of control cells stained with PI, DAPI and merged.

Action mechanism of AMPs is primarily membrane destabilization and disruption (Lin et al., 2022). The integrity of the bacterial membrane was examined in this study. *E. coli* was the experimental strain. The mechanism of action was proved by the following three experiments such as confocal laser scanning microscopy, membrane depolarization, and outer membrane permeabilization (Figures 6–8).

#### Cytoplasmic Membrane Depolarization

These compounds were able to dissipate the membrane of Gram-negative bacteria. The dye diSC35, membrane-potential fluorescence sensitive dye, was used in this experiment. The final concentration of the compounds was 6.25 μmol/L in the experiment. As illustrated in Figure 6, compound E6, E12 and E18 showed maximum membrane depolarization. As the number of arginine increased in compounds E1-E6, the membrane depolarization was enhanced trend correspondingly. It was further determined that biological activity of conjugates to dissipate the membrane-potential was positively correlated with their MIC values.

#### Outer Membrane Permeabilization

The hydrophobic dye N-phenyl naphthylamine (NPN) was used to study the outer membrane permeabilization. Normally, NPN was outside the outer membrane of Gram-negative bacteria. Nevertheless, bacterial outer membrane was damaged when uptake of NPN increased, and fluorescence intensity also increased. The final concentration of all the tested compounds was 6.25 μmol/L in the experiment. As illustrated in Figure 7, compound E6, E12 and E18 showed maximum outer membrane damage. As the number of arginine increased in compounds E1-E6, outer membrane damage was aggravated trend correspondingly. This indicated that compound E1-E6, E12 and E18 were able to permeate bacteria’s cell membrane.

#### Confocal Laser Scanning Microscopy

The dyes PI (propidium iodide) and DAPI (4′, 6-diamidino-2-phenylindole) were used in this experiment, and the experimental results were detected by confocal laser scanning microscopy (Figure 8). DAPI is a specific dye for DNA (blue) and PI is a fluorescent dye that intercalates into DNA (red) *via* a compromised cell membrane. Compound E6 displayed promising antibacterial activities and was further evaluated for membrane permeabilization by confocal laser scanning microscopy. After incubation of compound E6 with *E. coli* for 2 h, some *E. coli* cells were stained red by PI. However, *E. coli* cells untreated with compound E6 were stained blue by DAPI, and few of *E. coli* cells were stained by PI. The above results clearly indicated that E6 could break the bacterial cell membrane.

## Experimental Section

### Materials and Instrumentation

Solvents and reagents were purchased from commercial companies (Sinopharm, Aladdin, Macklin) and were of reagent grade. *E. coli* (ATCC 25922), *S. aureus* (ATCC 25923), *MRSA* (ATCC 43300) were obtained from American type culture collection. Clinical strains of *E. coli* (2019XJ06, 2019XJ25, 2018XJ108, 2018XJ105, 2018XJ30) and clinical strains of florfenicol resistant *E. coli* (2017XJ30, 2019XJ20) were collected from Lanzhou Institute of Husbandry and Pharmaceutical Sciences of CAAS. Fresh sterile defiber sheep blood was purchased from Beijing Land Bridge Technology Co., Ltd. in China. ^1^H NMR and ^13^C NMR spectra were recorded on a Bruker 400 and 101 MHz spectrometer, respectively. High resolution mass spectra (HRMS) were recorded on the Agilent Technologies 6530 Accurate-mass Q-TOF LC/MS. Analytical thin layer chromatography (TLC) plates were purchased from Qingdao Marine Chemical Factory in China. Column chromatography was performed on silica gel (300–400 meshes). Fluorescence measurements were obtained by multimode plate reader (PerkinElmer). Preparative reversed-phase high-performance liquid chromatography system (Pre - HPLC) was purchased from Hanbon sci. & Tech (Jiangsu, China).

### General Procedure for the Synthesis of Compounds (C1, C2, C3)

To the mixture of florfenicol (10 mmol) and succinic anhydride \ glutaric anhydride \ hexanedioic anhydride (15 mmol) in 25 ml acetone was added 4-dimethylaminopyridine (DMAP). DMAP was 4% of the quality of florfenicol. The compounds (C1, C2, C3) were obtained by reflux for 6 h. Upon completion, the solvent was removed by rotary evaporation at 50°C, and then saturated NaHCO_3_ solution at 50°C was slowly added until there were no bubbles in the solution. After filtering to remove impurities, 0.1 mol/L hydrochloric acid solution was slowly added to pH2.0, and the liquid was then crystallized at 4°C. After the precipitate was filtrated, compounds (C1, C2, C3) were obtained by washing and drying without further purification (Okuno et al., 2015).

Compound C1: ^1^H NMR (400 MHz, DMSO-*d*_6_) δ 12.33 (s, 1H), 8.91 (d, *J* = 8.8 Hz, 1H), 7.89 (d, *J* = 8.5 Hz, 2H), 7.61 (d, *J* = 8.4 Hz, 2H), 6.44 (s, 1H), 6.01 (d, *J* = 4.3 Hz, 1H), 4.64–4.29 (m, 3H), 3.20 (s, 3H), 2.68–2.62 (m, 2H), 2.57–2.52 (m, 2H).^13^C NMR (101 MHz, DMSO-*d*_6_) δ 173.45, 171.26, 163.96, 142.74, 140.48, 127.43, 127.00, 82.87, 81.18, 66.33, 53.20, 43.52, 28.93, 28.66. Chemical Formula: C_16_H_18_Cl_2_FNO_7_S, Exact Mass: 457.0165, HRMS (+TOF MS):475.0517 (M+NH_4_^+^).

Compound C2:^1^H NMR (400 MHz, DMSO-*d*_6_) δ ^1^H NMR (400 MHz, DMSO-*d*_6_) δ 12.16 (s, 1H), 8.95 (d, *J* = 8.8 Hz, 1H), 7.95–7.89 (m, 2H), 7.66–7.59 (m, 2H), 6.45 (s, 1H), 6.00 (d, *J* = 5.0 Hz, 1H), 4.61–4.28 (m, 3H), 3.21 (s, 3H), 2.50–2.43 (m, 2H), 2.24 (t, *J* = 7.3 Hz, 2H), 1.81–1.70 (m, 2H). ^13^C NMR (101 MHz, DMSO-*d*_6_) δ 174.01, 171.68, 163.95, 142.82, 140.62, 127.54, 127.17, 82.96, 66.34, 53.15, 43.52, 32.67, 19.75. Chemical Formula: C_17_H_20_Cl_2_FNO_7_S, Exact Mass: 471.0322, HRMS (+TOF MS):489.0721 (M+NH_4_^+^).

Compound C3: ^1^H NMR (400 MHz, DMSO-*d*_6_) δ 10.55 (s, 1H), 7.88 (d, *J* = 8.0 Hz, 2H), 7.66 (d, *J* = 8.0 Hz, 2H), 7.23 (s, 1H), 5.94 (d, *J* = 4.3 Hz, 1H), 4.67–4.47 (m, 1H), 4.48–4.24 (m, 2H), 3.19 (s, 3H), 2.42–2.31 (m, 2H), 2.01–1.92 (m, 2H), 1.59–1.49 (m, 4H), 1.23 (s, 2H). ^13^C NMR (101 MHz, DMSO-*d*_6_) δ 175.93, 171.93, 164.29, 143.22, 140.44, 127.59, 127.05, 82.78, 81.09, 66.69, 53.13, 43.52, 35.84, 33.30, 24.89, 24.10. Chemical Formula: C_18_H_22_Cl_2_FNO_7_S, Exact Mass: 485.0478, HRMS (+TOF MS):503.0818 (M+NH_4_^+^).

### Preparation of Polyarginine

The synthesis of polyarginine was used by the 9-fluorenylmethoxy carbonyl (Fmoc) solid-phase peptide synthesis method (Mäde et al., 2014). The Fmoc protecting group was deblocked with 25% piperidine by means of N,N-Dimethylformamide (DMF). The resin was washed three times with DMF, methanol, and dichloromethane after each coupling and deprotection step. To release the synthesized peptides from resins, 82.5% trifluoroacetic acid (TFA) was used in conjunction with appropriate scavengers. The crude polyarginines were purified on a Pre-HPLC with a Waters X-bridge C18, 5 μmol/L, 19 mm × 100 mm column. Peptide purities were detected on a HPLC system with a Waters X-bridge C18, 5 μmol/L, 4.6 mm × 250 mm column. Q-TOF LC/MS were used to determine the molecular mass of peptides.

Polyarginine D3: ^1^H NMR (400 MHz, Chloroform-*d*) δ 8.62 (d, *J* = 7.7 Hz, 1H), 8.26 (s, 2H), 8.07 (t, *J* = 5.7 Hz, 1H), 7.99 (t, *J* = 5.9 Hz, 1H), 7.62–7.29 (m, 7H), 7.14 (d, *J* = 2.1 Hz, 1H), 4.24 (s, 1H), 3.86 (t, *J* = 6.2 Hz, 1H), 3.17–3.01 (m, 4H), 1.76–1.65 (m, 3H), 1.59–1.42 (m, 5H). ^13^C NMR (101 MHz, CDCl_3_) δ 173.25, 172.40, 168.68, 160.29, 159.97, 159.65, 159.33, 157.39, 157.37, 121.66, 118.71, 115.75, 112.79, 52.68, 52.05, 40.61, 40.52, 29.50, 28.72, 25.46, 24.29, 21.38. Chemical Formula: C_12_H_27_N_9_O_2_, Exact Mass: 329.2288, HRMS (+TOF MS):330.2344 (M+H^+^).

Polyarginine D4: ^1^H NMR (400 MHz, Chloroform-*d*) δ 8.65 (d, *J* = 7.3 Hz, 1H), 8.24 (d, *J* = 7.7 Hz, 4H), 8.09–7.79 (m, 6H), 7.53–7.20 (m, 11H), 7.14 (s, 1H), 4.33 (q, *J* = 7.3, 6.7 Hz, 1H), 4.27–4.10 (m, 2H), 3.91–3.80 (m, 1H), 3.17–3.04 (m, 8H), 1.78–1.63 (m, 5H), 1.61–1.39 (m, 11H). ^13^C NMR (101 MHz, CDCl_3_) δ 173.70, 171.49, 171.32, 168.75, 160.22, 159.89, 159.56, 159.23, 157.32, 157.28, 121.38, 118.43, 115.49, 112.55, 52.80, 52.36, 52.04, 40.76, 40.60, 40.47, 29.53, 29.45, 29.33, 28.73, 25.39, 25.32, 24.36. Chemical Formula: C_24_H_51_N_17_O_4_, Exact Mass: 641.4310, HRMS (+TOF MS):642.4366 (M+H^+^).

Polyarginine D5: ^1^H NMR (400 MHz, Chloroform-*d*) δ 8.64 (d, *J* = 7.1 Hz, 1H), 8.24 (q, *J* = 12.3, 6.2 Hz, 4H), 8.13 (t, *J* = 7.8 Hz, 2H), 8.02–7.93 (m, 4H), 7.92–7.82 (m, 3H), 7.54–7.21 (m, 15H), 7.15 (s, 1H), 4.33 (d, *J* = 6.0 Hz, 1H), 4.23 (t, *J* = 7.0 Hz, 3H), 4.19–4.12 (m, 1H), 3.84 (q, *J* = 6.0 Hz, 1H), 3.09 (q, *J* = 6.0 Hz, 12H), 1.79–1.61 (m, 7H), 1.60–1.36 (m, 17H).^13^C NMR (101 MHz, CDCl_3_) δ 173.76, 171.76, 171.55, 171.37, 168.79, 160.42, 160.09, 159.77, 159.44, 157.38, 157.36, 157.31, 121.49, 118.54, 115.59, 112.65, 52.78, 52.61, 52.43, 52.08, 40.79, 40.62, 40.48, 29.50, 28.77, 25.41, 24.40. Chemical Formula: C_36_H_75_N_25_O_6_, Exact Mass: 953.6332, HRMS (+TOF MS):477.8222 (M+2H^+^)/2.

Polyarginine D6: ^1^H NMR (400 MHz, Chloroform-*d*) δ 8.63 (d, *J* = 7.1 Hz, 1H), 8.27–8.20 (m, 3H), 8.12 (q, *J* = 6.6 Hz, 3H), 8.00–7.79 (m, 9H), 7.54–7.21 (m, 19H), 7.14 (s, 1H), 4.33 (d, *J* = 6.5 Hz, 1H), 4.25 (d, *J* = 7.3 Hz, 5H), 4.16 (q, *J* = 7.3 Hz, 1H), 3.84 (q, *J* = 6.1 Hz, 1H), 3.30–2.82 (m, 16H), 1.83–1.62 (m, 9H), 1.60–1.28 (m, 23H). ^13^C NMR (101 MHz, CDCl_3_) δ 173.82, 171.80, 171.61, 171.43, 168.84, 160.37, 160.03, 159.70, 159.36, 157.40, 157.37, 157.34, 121.24, 118.31, 115.38, 112.45, 52.83, 52.61, 52.48, 52.13, 40.83, 40.66, 40.52, 28.80, 25.44, 24.44. Chemical Formula: C_48_H_99_N_33_O_8_, Exact Mass: 1265.8354, HRMS (+TOF MS):633.9237 (M+2H^+^)/2.

### General Procedure for the Synthesis of Conjugates (E1-E18)

The compounds C1-3 (0.065 mmol) and (3-Hydroxy-3H-1,2,3-triazolo [4,5-b]pyridinato-O)tri-1-pyrrolidinylphosphonium hexafluorophosphate (Pyaop, 0.078 mmol) were added to an oven-dried vial containing a stir bar (A). D1-6 were added to the vial (B). Both vials were filled with nitrogen and 0.5 ml dry DMF was added to each vial. Vial A was stirred at room temperature for 0.5 h and transferred to vial B. After adding N, N-Diisopropylethylamine (DIPEA, 0.13 mmol), the reaction system reacted at room temperature for 6 h. Upon completion, the solvent was lyophilized by a freeze dryer, and then the products were prepared by Pre-HPLC (Antonoplis et al., 2018).

Conjugate E1:^1^H NMR (400 MHz, DMSO-*d*_6_) δ 8.94 (d, *J* = 8.8 Hz, 1H), 8.48–8.19 (m, 1H), 7.96–7.81 (m, 2H), 7.76 (q, *J* = 6.5, 6.1 Hz, 1H), 7.61 (d, *J* = 8.1 Hz, 2H), 7.38 (s, 2H), 7.13 (d, *J* = 51.0 Hz, 2H), 6.46 (s, 1H), 6.00 (d, *J* = 4.0 Hz, 1H), 4.73–4.29 (m, 3H), 4.27–4.14 (m, 1H), 3.59 (s, 3H), 3.19 (s, 3H), 3.08 (q, *J* = 6.6 Hz, 2H), 2.64 (q, *J* = 6.8 Hz, 2H), 2.06 (s, 1H), 1.80–1.65 (m, 1H), 1.64–1.37 (m, 3H). ^13^C NMR (101 MHz, DMSO) δ 173.66, 172.66, 171.61, 171.57, 171.21, 171.04, 164.14, 159.57, 159.22, 158.88, 158.54, 157.05, 143.00, 140.60, 127.57, 127.17, 126.73, 120.82, 118.29, 117.90, 114.97, 112.05, 83.05, 81.36, 72.68, 72.61, 66.48, 53.40, 53.20, 52.04, 51.89, 51.76, 43.68, 40.48, 29.58, 29.16, 28.54, 28.32, 25.37, 25.25. Chemical Formula: C_23_H_32_Cl_2_FN_5_O_8_S, Exact Mass: 627.1333, HRMS (+TOF MS): 628.1397 (M+H^+^).

Conjugate E2:^1^H NMR (400 MHz, DMSO-*d*_6_) δ 8.93 (d, *J* = 8.8 Hz, 1H), 8.07 (d, *J* = 8.1 Hz, 1H), 7.94–7.85 (m, 2H), 7.73–7.64 (m, 1H), 7.63–7.58 (m, 2H), 7.37 (d, *J* = 2.1 Hz, 2H), 7.07 (d, *J* = 2.1 Hz, 2H), 6.46 (s, 1H), 6.00 (d, *J* = 4.1 Hz, 1H), 4.65–4.30 (m, 3H), 4.16 (s, 1H), 3.19 (s, 3H), 3.08 (q, *J* = 6.4 Hz, 2H), 7.72–7.57 (m, 2H), 2.07 (s, 2H), 1.75–1.60 (m, 1H), 1.54–1.37 (m, 3H). ^13^C NMR (101 MHz, DMSO) δ 173.69, 171.74, 170.88, 164.12, 158.98, 158.63, 158.29, 156.94, 142.96, 140.59, 127.56, 127.17, 118.29, 117.82, 114.90, 83.01, 81.32, 72.58, 72.52, 66.46, 53.37, 53.17, 52.16, 43.66, 40.60, 29.77, 29.41, 29.24, 25.36. Chemical Formula: C_22_H_31_Cl_2_FN_6_O_7_S Exact Mass: 612.1336, HRMS (+TOF MS):613.1412 (M+H^+^).

Conjugate E3:^1^H NMR (400 MHz, DMSO-*d*_6_) δ 8.96 (d, *J* = 8.8 Hz, 1H), 8.17 (d, *J* = 7.6 Hz, 1H), 7.97 (d, *J* = 7.9 Hz, 1H), 7.91–7.84 (m, 2H), 7.81–7.68 (m, 2H), 7.60 (d, *J* = 8.2 Hz, 2H), 7.33 (d, *J* = 2.1 Hz, 4H), 7.16–6.92 (m, 4H), 6.47 (s, 1H), 5.99 (d, *J* = 4.2 Hz, 1H), 4.72–4.40 (m, 3H), 4.36–4.21 (m, 1H), 4.19–4.11 (m, 1H), 3.20 (s, 3H), 3.07 (q, *J* = 6.5 Hz, 4H), 2.71–2.57 (m, 2H), 2.47 (d, *J* = 8.0 Hz, 2H), 1.64 (q, *J* = 17.3 Hz, 2H), 1.54–1.37 (m, 6H). ^13^C NMR (101 MHz, DMSO) δ 173.43, 171.72, 171.58, 171.16, 164.13, 162.53, 159.18, 158.86, 158.53, 156.99, 142.91, 140.61, 127.55, 127.19, 118.44, 115.49, 83.00, 81.31, 72.69, 72.62, 66.46, 53.35, 53.15, 52.53, 52.17, 43.66, 40.66, 40.54, 36.01, 29.72, 29.44, 29.24, 29.21, 25.32, 25.18. Chemical Formula: C_28_H_43_Cl_2_FN_10_O_8_S, Exact Mass: 768.2347, HRMS (+TOF MS):769.2391 (M+H^+^).

Conjugate E4:^1^H NMR (400 MHz, DMSO-*d*_6_) δ 8.98 (d, *J* = 8.7 Hz, 1H), 8.14 (d, *J* = 7.7 Hz, 1H), 8.07 (q, *J* = 17.5 Hz, 2H), 7.94 (d, *J* = 5.4 Hz, 1H), 7.89 (d, *J* = 8.3 Hz, 2H), 7.82 (t, *J* = 5.8 Hz, 1H), 7.77–7.68 (m, 3H), 7.60 (d, *J* = 8.2 Hz, 2H), 7.55–7.30 (m, 7H), 7.14 (s, 7H), 6.47 (s, 1H), 5.99 (d, *J* = 4.2 Hz, 1H), 4.63–4.41 (m, 3H), 4.36–4.18 (m, 3H), 4.15 (t, *J* = 7.0 Hz, 1H), 3.19 (s, 3H), 3.07 (q, *J* = 6.6 Hz, 3H), 2.70–2.55 (m,2H), 2.46 (d, *J* = 8.3 Hz, 2H), 1.77–1.59 (m, 4H), 1.47 (m, 12H). ^13^C NMR (101 MHz, DMSO) δ 173.55, 171.84, 171.70, 171.54, 171.34, 171.14, 164.16, 162.55, 159.84, 159.51, 159.18, 158.86, 157.06, 142.94, 140.64, 127.59, 127.19, 121.35, 118.40, 115.46, 112.51, 82.99, 81.31, 72.70, 66.48, 53.37, 53.18, 52.56, 52.40, 52.25, 43.66, 40.67, 40.54, 36.01, 31.00, 29.72, 29.43, 29.21, 25.29, 25.24, 25.14. Chemical Formula: C_40_H_67_Cl_2_FN_18_O_10_S, Exact Mass: 1080.4369, HRMS (+TOF MS):541.2231 (M+2H^+^)/2.

Conjugate E5:^1^H NMR (400 MHz, DMSO-*d*_6_) δ 8.98 (d, *J* = 8.8 Hz, 1H), 8.24–8.01 (m, 5H), 7.97 (d, *J* = 7.5 Hz, 1H), 7.89 (d, *J* = 8.1 Hz, 2H), 7.79 (t, *J* = 5.8 Hz, 1H), 7.71 (t, *J* = 6.5 Hz, 5H), 7.60 (d, *J* = 8.1 Hz, 2H), 7.52–7.27 (m, 10H), 7.10 (t, *J* = 19.7 Hz, 10H), 6.47 (s, 1H), 5.99 (d, *J* = 4.3 Hz, 1H), 4.63–4.31 (m, 3H), 4.24 (q, *J* = 6.8 Hz, 5H), 4.15 (q, *J* = 7.1 Hz, 1H), 3.19 (s, 3H), 3.14–2.94 (m, 12H), 2.74–2.56 (m, 2H), 2.46 (d, *J* = 9.9 Hz, 2H), 1.66 (q, *J* = 16.9 Hz, 6H), 1.50 (m, 18H). ^13^C NMR (101 MHz, DMSO) δ 173.63, 171.94, 171.75, 171.61, 171.43, 171.27, 164.22, 160.06, 159.72, 159.38, 159.05, 157.10, 142.98, 140.69, 127.65, 127.25, 121.08, 118.15, 115.21, 112.28, 83.03, 81.34, 72.72, 66.52, 53.41, 53.22, 52.65, 43.70, 40.73, 40.60, 29.77, 29.47, 29.22, 25.34. Chemical Formula: C_52_H_91_Cl_2_FN_26_O_12_S, Exact Mass: 1392.6392, HRMS (+TOF MS):697.3195 (M+2H^+^)/2.

Conjugate E6:^1^H NMR (400 MHz, DMSO-*d*_6_) δ 9.00 (d, *J* = 8.8 Hz, 1H), 8.20–8.03 (m, 7H), 7.99 (d, *J* = 7.4 Hz, 1H), 7.90 (d, *J* = 8.2 Hz, 2H), 7.86–7.82 (m, 1H), 7.77 (d, *J* = 6.1 Hz, 7H), 7.61 (d, *J* = 8.2 Hz, 2H), 7.53–7.32 (m, 13H), 7.23–7.07 (m, 13H), 6.48 (s, 1H), 6.00 (d, *J* = 4.2 Hz, 1H), 4.52–4.32 (m, 3H), 4.25 (s, 7H), 4.19–4.12 (m, 1H), 3.19 (s, 3H), 3.14–3.03 (m, 16H), 2.71–2.59 (m, 2H), 2.47 (d, *J* = 10.0 Hz, 2H), 1.67 (d, *J* = 10.5 Hz, 8H), 1.57–1.42 (m, 24H). ^13^C NMR (101 MHz, DMSO) δ 173.67, 172.00, 171.77, 171.66, 171.48, 171.32, 164.25, 160.27, 159.94, 159.61, 159.28, 157.16, 143.02, 140.69, 127.65, 127.26, 121.31, 118.37, 115.42, 112.48, 83.05, 81.35, 72.73, 66.54, 53.45, 53.25, 52.71, 52.44, 52.39, 43.72, 40.74, 40.61, 29.79, 29.48, 29.26, 25.30, 22.73. Chemical Formula: C_64_H_115_Cl_2_FN_34_O_14_S, Exact Mass: 1704.8414, HRMS (+TOF MS):854.4191 (M+2H^+^)/2.

Conjugate E7:^1^H NMR (400 MHz, DMSO-*d*_6_) δ 9.02 (d, *J* = 8.9 Hz, 1H), 8.28 (d, *J* = 7.3 Hz, 1H), 7.97–7.86 (m, 2H), 7.79 (s, 1H), 7.62 (d, *J* = 8.2 Hz, 2H), 7.40 (s, 2H), 7.09 (s, 2H), 6.48 (s, 1H), 6.00 (d, *J* = 4.8 Hz, 1H), 4.63–4.27 (m, 3H), 4.26–4.14 (m, 1H), 3.62 (s, 3H), 3.19 (s, 3H), 3.09 (q, *J* = 6.7 Hz, 2H), 2.47–2.37 (m, 2H), 2.17 (t, *J* = 7.3 Hz, 2H), 1.84–1.65 (m, 3H), 1.64–1.42 (m, 3H). ^13^C NMR (101 MHz, DMSO) δ 173.80, 172.78, 172.13, 171.99, 171.90, 164.17, 159.70, 159.34, 159.00, 158.67, 157.10, 143.07, 140.73, 127.67, 127.33, 126.75, 118.28, 117.94, 115.02, 83.09, 81.40, 72.77, 72.71, 66.51, 53.34, 53.15, 52.04, 51.86, 51.73, 43.67, 40.49, 34.11, 33.99, 32.92, 28.36, 28.15, 25.46, 25.33, 20.53. Chemical Formula: C_24_H_34_Cl_2_FN_5_O_8_S, Exact Mass: 641.1489, HRMS (+TOF MS):642.1572 (M+H^+^).

Conjugate E8:^1^H NMR (400 MHz, DMSO-*d*_6_) δ 9.01 (d, *J* = 8.8 Hz, 1H), 7.96 (d, *J* = 8.1 Hz, 1H), 7.93–7.87 (m, 2H), 7.72 (t, *J* = 5.7 Hz, 1H), 7.65–7.58 (m, 2H), 7.40 (d, *J* = 2.1 Hz, 2H), 7.06 (d, *J* = 2.1 Hz, 2H), 6.47 (s, 1H), 5.99 (d, *J* = 4.8 Hz, 1H), 4.61–4.27 (m, 3H), 4.24–4.14 (m, 1H), 3.20 (s, 3H), 3.08 (q, *J* = 6.5 Hz, 2H), 2.48–2.38 (m, 2H), 2.23–2.10 (m, 2H), 1.74 (q, *J* = 13.6 Hz, 2H), 1.70–1.61 (m, 1H), 1.55–1.38 (m, 3H).^13^C NMR (101 MHz, DMSO) δ 173.80, 171.91, 171.73, 164.14, 159.16, 158.83, 157.00, 143.02, 140.70, 127.64, 127.30, 83.08, 81.38, 72.73, 72.67, 66.49, 53.31, 53.12, 52.08, 45.95, 45.90, 43.65, 40.59, 34.21, 33.07, 29.39, 25.40, 20.56. Chemical Formula: C_23_H_33_Cl_2_FN_6_O_7_S, Exact Mass: 626.1493, HRMS (+TOF MS):627.1574 (M+H^+^).

Conjugate E9:^1^H NMR (400 MHz, DMSO-*d*_6_) δ 9.01 (d, *J* = 8.8 Hz, 1H), 8.06 (d, *J* = 7.6 Hz, 1H), 7.96 (d, *J* = 7.9 Hz, 1H), 7.93–7.88 (m, 2H), 7.81–7.66 (m, 2H), 7.65–7.58 (m, 2H), 7.52–7.26 (m, 4H), 7.20–6.96 (m, 4H), 6.48 (s, 1H), 5.99 (d, *J* = 4.9 Hz, 1H), 4.63–4.38 (m, 3H), 4.34–4.14 (m, 2H), 3.20 (s, 3H), 3.14–3.01 (m, 4H), 2.48–2.39 (m, 2H), 2.17 (t, *J* = 7.4 Hz, 2H), 1.80–1.60 (m, 4H), 1.57–1.37 (m, 6H). ^13^C NMR (101 MHz, DMSO) δ 173.44, 172.02, 171.88, 171.67, 164.13, 159.55, 159.23, 158.91, 158.59, 157.00, 142.98, 140.71, 127.65, 127.29, 121.44, 118.48, 115.53, 112.57, 83.04, 81.35, 72.75, 72.69, 66.48, 53.29, 53.10, 52.48, 52.11, 43.63, 40.66, 40.54, 34.20, 33.05, 29.36, 29.26, 25.30, 25.24, 20.57. Chemical Formula: C_29_H_45_Cl_2_FN_10_O_8_S, Exact Mass: 782.2504, HRMS (+TOF MS):783.2544 (M+H^+^).

Conjugate E10:^1^H NMR (400 MHz, DMSO-*d*_6_) δ 9.00 (d, *J* = 8.8 Hz, 1H), 8.06 (q, *J* = 11.5 Hz, 3H), 7.98–7.93 (m, 1H), 7.93–7.88 (m, 2H), 7.70 (d, *J* = 5.7 Hz, 1H), 7.66 (d, *J* = 5.5 Hz, 2H), 7.61 (d, *J* = 8.4 Hz, 3H), 7.41 (d, *J* = 46.0 Hz, 7H), 7.15 (s, 7H), 6.47 (s, 1H), 5.98 (d, *J* = 5.0 Hz, 1H), 4.64–4.29 (m, 3H), 4.28–4.20 (m, 3H), 4.15 (q, *J* = 7.2 Hz, 1H), 3.20 (s, 3H), 3.08 (t, *J* = 6.7 Hz, 8H), 2.47–2.36 (m, 2H), 2.23–2.09 (m, 2H), 1.74 (t, *J* = 7.7 Hz, 2H), 1.70–1.61 (m, 4H), 1.49 (q, *J* = 23.8 Hz, 12H). ^13^C NMR (101 MHz, DMSO) δ 173.53, 172.11, 171.93, 171.89, 171.55, 171.33, 164.16, 162.56, 159.85, 159.52, 159.19, 158.85, 156.97, 142.96, 140.76, 127.70, 127.33, 118.21, 115.27, 83.05, 81.36, 72.76, 66.50, 53.31, 53.12, 52.52, 52.41, 52.35, 52.26, 43.64, 40.69, 40.59, 36.02, 34.23, 33.07, 31.00, 29.45, 29.31, 25.30, 25.21, 25.12, 20.56. Chemical Formula: C_41_H_69_Cl_2_FN_18_O_10_S, Exact Mass: 1094.4526, HRMS (+TOF MS):548.2311 (M+2H^+^)/2.

Conjugate E11:^1^H NMR (400 MHz, DMSO-*d*_6_) δ 9.04 (d, *J* = 8.8 Hz, 1H), 8.17–8.01 (m, 5H), 7.97 (d, *J* = 7.6 Hz, 1H), 7.90 (d, *J* = 8.3 Hz, 2H), 7.88–7.84 (m, 1H), 7.78 (q, *J* = 7.4, 6.4 Hz, 5H), 7.61 (d, *J* = 8.2 Hz, 2H), 7.53–7.30 (m, 10H), 7.28–7.00 (m, 10H), 6.49 (s, 1H), 5.98 (d, *J* = 4.8 Hz, 1H), 4.63–4.29 (m, 3H), 4.24 (t, *J* = 7.2 Hz, 5H), 4.15 (q, *J* = 7.2 Hz, 1H), 3.19 (s, 3H), 3.13–3.02 (m, 12H), 2.47–2.35 (m, 2H), 2.24–2.10 (m, *J* = 7.1 Hz, 2H), 1.75 (q, *J* = 7.6 Hz, 2H), 1.65 (m, 4H), 1.57–1.40 (m, 18H). ^13^C NMR (101 MHz, DMSO) δ 173.58, 172.19, 172.02, 171.89, 171.61, 171.59, 171.40, 164.17, 160.02, 159.70, 159.37, 159.05, 157.11, 143.01, 140.72, 127.66, 127.30, 121.40, 118.45, 115.50, 112.55, 83.04, 81.35, 72.72, 66.50, 53.31, 53.12, 52.62, 52.50, 52.35, 43.65, 40.67, 34.24, 33.06, 25.31, 25.21, 20.54. Chemical Formula: C_53_H_93_Cl_2_FN_26_O_12_S, Exact Mass: 1406.6548, HRMS (+TOF MS):704.3287 (M+2H^+^)/2.

Conjugate E12:^1^H NMR (400 MHz, DMSO-*d*_6_) δ 9.05 (d, *J* = 8.8 Hz, 1H), 8.21–8.02 (m,7H), 7.98 (d, *J* = 7.4 Hz, 1H), 7.90 (d, *J* = 8.3 Hz, 2H), 7.88–7.84 (m, 1H), 7.82–7.74 (m, 7H), 7.61 (d, *J* = 8.2 Hz, 2H), 7.55–7.32 (m, 13H), 7.26–7.05 (m, 13H), 6.49 (s, 1H), 5.98 (d, *J* = 4.8 Hz, 1H), 4.64–4.30 (m, 3H), 4.27–4.20 (m, 7H), 4.15 (q, *J* = 7.2 Hz, 1H), 3.19 (s, 3H), 3.15–2.92 (m, 16H), 2.48–2.33 (m, 2H), 2.25–2.09 (m, 2H), 1.78–1.61 (m, 10H), 1.57–1.40 (m, 24H). ^13^C NMR (101 MHz, DMSO) δ 173.67, 172.31, 172.13, 171.96, 171.71, 171.67, 171.48, 164.24, 160.16, 159.82, 159.49, 159.15, 157.17, 143.08, 140.77, 127.72, 127.36, 121.18, 118.25, 115.31, 112.38, 83.09, 81.40, 72.84, 72.78, 66.55, 53.38, 53.18, 52.71, 52.61, 52.43, 43.70, 40.74, 40.71, 40.60, 34.29, 33.10, 29.46, 29.23, 20.60. Chemical Formula: C_65_H_117_Cl_2_FN_34_O_14_S, Exact Mass: 1718.8570, HRMS (+TOF MS):861.4254 (M+2H^+^)/2.

Conjugate E13:^1^H NMR (400 MHz, DMSO-*d*_6_) δ 8.99 (d, *J* = 8.9 Hz, 1H), 8.27 (d, *J* = 7.4 Hz, 1H), 7.96–7.88 (m, 2H), 7.72 (t, *J* = 5.6 Hz, 1H), 7.65–7.57 (m, 2H), 7.36 (s, 2H), 7.11 (d, *J* = 51.0 Hz, 2H), 6.48 (s, 1H), 5.99 (d, *J* = 4.9 Hz, 1H), 4.64–4.27 (m, 3H), 4.26–4.18 (m, 1H), 3.60 (s, 3H), 3.20 (s, 3H), 3.08 (q, *J* = 6.6 Hz, 2H), 2.42 (t, *J* = 6.9 Hz, 2H), 2.13 (t, *J* = 6.7 Hz, 2H), 1.77–1.66 (m, 1H), 1.63–1.43 (m, 7H). ^13^C NMR (101 MHz, DMSO) δ 173.51, 172.50, 172.22, 171.76, 163.85, 159.24, 158.92, 158.59, 158.27, 156.73, 142.76, 140.44, 127.40, 127.03, 118.13, 115.17, 82.79, 81.10, 72.44, 72.38, 66.23, 53.04, 52.84, 51.75, 51.50, 43.38, 40.19, 34.41, 33.12, 28.98, 27.90, 25.04, 24.52, 23.73. Chemical Formula: C_25_H_36_Cl_2_FN_5_O_8_S, Exact Mass: 655.1646, HRMS (+TOF MS):656.1743 (M+H^+^).

Conjugate E14:^1^H NMR (400 MHz, DMSO-*d*_6_) δ 9.00 (d, *J* = 8.8 Hz, 1H), 7.98–7.91 (m, 2H), 7.90 (d, *J* = 1.8 Hz, 1H), 7.70 (t, *J* = 5.8 Hz, 1H), 7.65–7.59 (m,2H), 7.39 (d, *J* = 2.2 Hz, 2H), 7.14–6.99 (m, 2H), 6.48 (s, 1H), 5.98 (d, *J* = 4.9 Hz, 1H), 4.62–4.25 (m, 3H), 4.24–4.14 (m, 1H), 3.20 (s, 3H), 3.08 (q, *J* = 6.4 Hz, 2H), 2.41 (t, *J* = 6.9 Hz, 2H), 2.14 (t, *J* = 6.8 Hz, 2H), 1.71–1.60 (m, 1H), 1.56–1.38 (m, 7H). ^13^C NMR (101 MHz, DMSO) δ 173.80, 172.14, 172.04, 164.11, 159.47, 159.14, 158.81, 158.49, 156.97, 143.02, 140.70, 127.66, 127.29, 118.31, 115.36, 83.04, 81.35, 72.69, 72.63, 66.50, 53.30, 53.10, 52.00, 43.64, 40.57, 34.87, 33.39, 29.44, 25.38, 24.82, 24.09. Chemical Formula: C_24_H_35_Cl_2_FN_6_O_7_S, Exact Mass (+TOF MS): 640.1649, HRMS (+TOF MS):641.1744 (M+H^+^).

Conjugate E15:^1^H NMR (400 MHz, DMSO-*d*_6_) δ 9.01 (d, *J* = 8.8 Hz, 1H), 8.05 (d, *J* = 7.6 Hz, 1H), 7.97 (d, *J* = 8.0 Hz, 1H), 7.95–7.88 (m, 2H), 7.64 (dd, *J* = 14.8, 7.7 Hz, 4H), 7.51–7.27 (m, 4H), 7.23–6.96 (m, 4H), 6.49 (s, 1H), 5.98 (d, *J* = 5.0 Hz, 1H), 4.63–4.37 (m, 3H), 4.35–4.13 (m, 2H), 3.21 (s, 3H), 3.08 (q, *J* = 13.0 Hz, 4H), 2.42 (t, *J* = 6.9 Hz, 2H), 2.20–2.09 (m, 2H), 1.71–1.60 (m, 2H), 1.55–1.43 (m, 9H), 1.28–1.20 (m, 1H). ^13^C NMR (101 MHz, DMSO) δ 173.42, 172.43, 172.04, 171.68, 164.12, 159.56, 159.23, 158.90, 158.58, 156.94, 142.98, 140.73, 127.69, 127.30, 118.34, 115.39, 83.02, 81.33, 72.72, 72.66, 66.50, 53.30, 53.10, 52.40, 52.09, 43.64, 40.66, 40.57, 34.86, 33.35, 29.38, 29.28, 25.28, 25.24, 24.84, 24.08. Chemical Formula: C_30_H_47_Cl_2_FN_10_O_8_S, Exact Mass: 796.2660, HRMS (+TOF MS):797.2745 (M+H^+^).

Conjugate E16:^1^H NMR (400 MHz, DMSO-*d*_6_) δ 9.01 (d, *J* = 8.8 Hz, 1H), 8.05 (q, *J* = 11.1 Hz, 3H), 7.94 (d, *J* = 7.6 Hz, 1H), 7.93–7.88 (m, 2H), 7.74 (d, *J* = 5.1 Hz, 1H), 7.71–7.63 (m, 3H), 7.61 (d, *J* = 8.4 Hz, 2H), 7.49–7.26 (m, 7H), 7.09 (d, *J* = 41.7 Hz, 7H), 6.48 (s, 1H), 5.97 (d, *J* = 5.1 Hz, 1H), 4.61–4.36 (m, 3H), 4.28–4.19 (m, 3H), 4.15 (d, *J* = 6.7 Hz, 1H), 3.20 (s, 3H), 3.07 (q, *J* = 6.7 Hz, 8H), 2.41 (t, *J* = 7.0 Hz, 2H), 2.23–2.18 (m, 1H), 2.13 (d, *J* = 7.2 Hz, 1H), 1.70–1.62 (m, 3H), 1.56–1.41 (m, 16H), 1.23 (q, *J* = 9.9 Hz, 1H). ^13^C NMR (101 MHz, DMSO) δ 174.54, 173.50, 173.43, 172.52, 172.04, 171.94, 171.52, 171.31, 164.12, 159.37, 159.04, 156.98, 142.96, 140.75, 127.70, 127.30, 118.32, 115.38, 83.01, 81.31, 72.67, 66.50, 53.29, 53.10, 52.51, 52.34, 52.24, 51.42, 43.63, 40.66, 34.87, 33.60, 33.51, 33.33, 33.20, 29.42, 29.29, 25.27, 25.18, 25.10, 24.83, 24.24, 24.16, 24.14, 24.09. Chemical Formula: C_42_H_71_Cl_2_FN_18_O_10_S, Exact Mass: 1108.4682, HRMS (+TOF MS):555.2412 (M+2H^+^)/2.

Conjugate E17:^1^H NMR (400 MHz, DMSO-*d*_6_) δ 9.01 (d, *J* = 8.8 Hz, 1H), 8.24–8.00 (m, 5H), 7.97 (d, *J* = 7.6 Hz, 1H), 7.91 (d, *J* = 8.3 Hz, 2H), 7.75–7.65 (m, 6H), 7.61 (d, *J* = 8.2 Hz, 2H), 7.54–7.25 (m, 10H), 7.09 (d, *J* = 52.4 Hz, 10H), 6.48 (s, 1H), 5.97 (d, *J* = 5.2 Hz, 1H), 4.63–4.29 (m, 3H), 4.23 (t, *J* = 6.2 Hz, 5H), 4.15 (q, *J* = 7.1 Hz, 1H), 3.20 (s, 3H), 3.13–3.00 (m, 12H), 2.41 (t, *J* = 7.0 Hz, 2H), 2.20 (t, *J* = 6.9 Hz, 1H), 2.17–2.06 (m, 1H), 1.64 (t, *J* = 8.8 Hz, 4H), 1.58–1.37 (m, 23H), 1.27–1.20 (m, 1H) ^13^C NMR (101 MHz, DMSO) δ 174.56, 174.51, 173.55, 173.44, 172.64, 172.05, 172.01, 171.56, 171.54, 171.36, 164.14, 159.99, 159.66, 159.33, 159.00, 157.00, 142.97, 140.77, 127.72, 127.32, 121.30, 118.35, 115.40, 112.46, 83.01, 81.32, 72.75, 66.52, 53.31, 53.11, 52.58, 52.31, 51.43, 43.64, 40.68, 34.91, 33.61, 33.52, 33.33, 33.22, 29.39, 25.30, 25.19, 24.84, 24.25, 24.17, 24.11. Chemical Formula: C_54_H_95_Cl_2_FN_26_O_12_S, Exact Mass: 1420.6705, HRMS (+TOF MS):711.3409 (M+2H^+^)/2.

Conjugate E18:^1^H NMR (400 MHz, DMSO-*d*_6_) δ 9.01 (d, *J* = 8.8 Hz, 1H), 8.16–8.00 (m, 7H), 7.97 (d, *J* = 7.4 Hz, 1H), 7.91 (d, *J* = 8.3 Hz, 2H), 7.75–7.65 (m, 8H), 7.61 (d, *J* = 8.3 Hz, 2H), 7.44 (d, *J* = 49.5 Hz, 13H), 7.11 (d, *J* = 37.1 Hz, 13H), 6.48 (s, 1H), 5.97 (d, *J* = 5.1 Hz, 1H), 4.59–4.40 (m, 3H), 4.25 (q, *J* = 7.0 Hz, 7H), 4.15 (q, *J* = 7.1 Hz, 1H), 3.19 (s, 3H), 3.14–2.96 (m, 16H), 2.41 (t, *J* = 7.0 Hz, 2H), 2.22–2.18 (m, 1H), 2.12 (q, *J* = 24.8 Hz, 1H), 1.67–1.61 (m, 8H), 1.56–1.39 (m, 28H).^13^C NMR (101 MHz, DMSO) δ 174.61, 174.57, 173.62, 173.49, 172.75, 172.10, 171.65, 171.61, 171.43, 164.19, 160.05, 159.71, 159.37, 159.03, 157.07, 157.06, 143.03, 140.80, 127.75, 127.36, 121.01, 118.08, 115.15, 112.22, 83.05, 81.36, 72.80, 72.74, 66.55, 53.36, 53.16, 52.65, 52.37, 51.45, 43.68, 40.75, 40.70, 40.62, 34.95, 33.65, 33.57, 33.37, 33.26, 29.47, 25.34, 25.25, 24.88, 24.29, 24.21, 24.20, 24.15. Chemical Formula: C_66_H_119_Cl_2_FN_34_O_14_S, Exact Mass: 1732.8727, HRMS (+TOF MS):868.4414 (M+2H^+^)/2.

### Bacterial Strains and Culture Conditions

Gram-negative *E. coli* (ATCC 25922), clinical strains of *E. coli* (2019XJ06, 2019XJ25, 2018XJ108, 2018XJ105, 2018XJ30) and clinical strains of florfenicol resistant *E. coli* (2017XJ30, 2019XJ20) and Gram-positive *MRSA* (ATCC 43300) and *S. aureus* (ATCC 25923) were maintained in 50% glycerol at −80°C up to the time of use. The bacterial strains were cultured in Muller-Hinton broth (Guangdong Huan Kai Microbial Sci. &Tech. Co., Ltd. in China) media. Mueller-Hinton agar (Guangdong Huan Kai Microbial Sci. & Tech. Co., Ltd. in China) was used as growth medium in solid media.

### Antibacterial Assay

CLSI guidelines were followed for determining MICs of all florfenicol-polyarginine conjugates by the broth microdilution method. The required bacterial (*E. coli*, *S. aureus and MRSA*) single colony was inoculated into Muller-Hinton broth (MHB) and cultured for 4–6 h. And then, the bacteria were adjusted to 10^6^ CFU/ml in cultures. All compounds were prepared into a stock solution at a concentration of 0.1 mol/L with DMSO. The stock solutions were then diluted to required concentration (100, 50, 25, 12.5, 6.25, 3.125, 1.56, 0.78, 0.39 μmol/L) with MHB media. Subsequently, a 96 well plate was filled with these dilutions and 10^6^ CFU/ml bacterial suspension. The diluted compounds of different concentrations (100 µl) were first added to 96 well plate, and then 100 µl bacterial dilutions (10^6^ CFU/ml) was added. The negative and positive controls were 200 µl of MHB media and 100 µl of bacterial suspension combined with 100 µl of MHB media, respectively. The experiment of each compound was repeated three times, and each experiment was at least twice repeated. The 96 well plates were incubated at 37°C for 16–24 h. MIC indicated antibacterial activity. Florfenicol was used as a control drug.

### Hemolytic Assay

The sheep erythrocytes were used to determine hemolytic activity of compounds. Red blood cells isolated from sheep blood were resuspended in 1×PBS (5%). These compounds (C1, D1-D6, E1-E6, E12 and E18) were serially diluted with distilled water into solutions of the different concentrations (1000, 900, 800, 700, 600, 500, 400, 300, 200, 100, 50, 25 μmol/L). Then red blood cells suspension (150 µL) and compound solutions (50 µl) were sequentially added in a 96-well plate. The negative and positive controls were 150 µl of red blood cells suspension combined with 50 µL distilled water and 50 µl 0.1% Triton X-100 solution, respectively. The plate incubated for 1 h at 37°C was centrifuged for 5 min at 2500 rpm. Supernatant from each well into a new plate was detected at 540 nm by the microplate reader. Erythrocyte hemolysis rate = (A − A _negative_) / (A _positive_ − A _negative_) × 100%, A is the absorbance of the test well.

### Cell Culture

The human colon carcinoma cell line (Caco-2) cells were cultured in 20% fetal bovine serum (FBS), 1% gluta-max, 1% sodium pyruvate, 1% non-essential amino acids (NEAA), 77% Modified Eagle Medium (MEM) media supplemented with 2 mM L-glutamine at 37°C under humidified atmospheric conditions containing 5% CO_2_.

### Cytotoxicity Assay

Cell Counting Kit (CCK-8) assays were used to determine cytotoxicity. In short, 5.0 × 10^3^ cells per well in 100 µl medium were seeded to every well of 96-well plates and incubated 24 h at 37°C. After removing culture medium, the cells were replaced with fresh medium (100 µl) containing different concentrations of compounds (E6, E12, E18). Negative control was culture medium containing corresponding concentration of DMSO. After treating for 24 h and removing culture medium, the cells were washed twice with PBS and added to 100 µl new medium with 5% CCK-8. The plate was incubated for 2 h at 37°C and detected at 540 nm by the microplate reader. The average 50% inhibitory concentration (IC_50_) was calculated by SPSS. Each concentration was tested three times.

### Propensity of Bacterial Resistance Development

Compound E6 was used to evaluate the tendency of developing bacterial resistance towards the florfenicol-polyarginine conjugates. The control antibiotic florfenicol was chosen for E. coli. MIC of E6 and florfenicol for 24 h were determined against E. coli., and then 0.5 MIC of E6 and florfenicol was challenged repeatedly against E. coli. After the initial MIC experiment, serial passage was investigated by transferring the growing bacterial suspension at sub-MIC of the compounds (MIC/2) and MIC in every passage was determined again. The process was repeated for 16 passages. The MICs for E6 and florfenicol corresponding to days were draw into figure to determine the propensity of bacterial resistance development.

### Antibacterial Activity in Plasma (Plasma Stability)

The fresh sterile and defibered sheep blood was centrifuged (3500 rpm, 10 min) at 4°C and the supernatant was harvested for the experiment. *E. coli* was cultured for 4–6 h in the manner mentioned in bacterial strains and culture conditions and then diluted to 10^5^ CFU/ml in the MHB media. The test compounds (E6, E12, E18) were dissolved in plasma/sterile water (v/v = 1:1) at different concentrations (100, 50, 25, 12.5, 6.25, 3.125, 1.56 μmol/L) and pre-incubated for 0, 3 and 6 h, respectively. Afterwards 50 µl of the above pre-incubated solutions and the bacterial suspension (150 μl, 10^5^ CFU/ml) were transferred into the wells of a 96-well plate. At 37°C, the plate was incubated for 20–24 h, 20 µl of which was plated and incubated for 20–24 h at 37°C. The MBC of each compound was the concentration without bacterial growth.

### Antibacterial Assay in Complex Mammalian Fluids

The fresh sterile and defibered sheep blood was purchased from Beijing Land Bridge Technology Co., Ltd. in China. The plasma was obtained according to the above description. Fetal bovine serum was bought from Gibco Life Technologies in America. *E. coli* was cultured for 4–6 h in the manner mentioned in bacterial strains and culture conditions, and then diluted to 10^5^ CFU/ml in the media with 50% MHB media and 50% mammalian media (serum, plasma, blood). The test compounds (E6, E12, E18) were dissolved in plasma/sterile water (v/v = 1:1) at different concentrations (100, 50, 25, 12.5, 6.25, 3.125, 1.56 μmol/L) and pre-incubated for 0, 3 and 6 h, respectively. Afterwards 50 µl of the above pre-incubated solutions and the bacterial suspension (150 μl, 10^5^ CFU/ml) were transferred into the wells of a 96-well plate. At 37°C, the plate was incubated for 20–24 h, 20 µl of which was plated and incubated for 20–24 h at 37°C. The MBC of each compound was the concentration without bacterial growth.

### Time-dependent Killing

*E. coli* (ATCC 25922) was cultured in MHB for 6 h at 37°C, and then the bacterial were diluted to approximately 6 × 10^5^ CFU/ml. Experimental compound E6 (1/2 MIC, 1 MIC, 2 MIC and 4 MIC) were inoculated into the bacterial suspension. The negative and positive controls were MHB media and florfenicol (4 × MIC), respectively. 0.5 ml aliquot took at different time point (0, 2, 4, 6, 8, 12 and 24 h) was serially diluted to 10-1 to 10-8 by 10-fold in 0.9% saline. The dilutions were then plated on sterile Muellere Hinton agar plates and incubated for 24 h at 37°C. The viable colonies were counted and represented as log10 (CFU/ml). The experiment was repeated three times for each concentration.

### Cytoplasmic Membrane Depolarization Assay

The 6 h grown *E. coli* was centrifuged (3500 rpm, 5 min) and resuspended at 4°C, washed and resuspended with 1 × PBS. This process was repeated twice. 150 µl *E. coli* suspension (−10^8^ CFU/ml) was then added to the wells of a 96-well black plate with clear bottom. Then 50 µl of 10 µM 3,3′- dipropylthiadicarbocyanine iodide (diSC35) and 50 µl of 200 µM EDTA were transferred to the wells and pre-incubated for 40 min. The fluorescence (622 nm/670 nm) was monitored every 2 min for 8 min. Then *E. coli* suspensions were added with 10 µl compound solution with E1, E2, E3, E4, E5, D5, E6, E12, E18, FFC and their final concentrations were 6.25 µM. Fluorescence was detected immediately for another 12 min at every 2 min interval after adding compounds. The control group was the same as that of the experimental group except that the solvent without compound was added.

### Outer Membrane Permeabilization Assay

The outer membrane permeabilization activity of the compounds (E1, E2, E3, E4, E5, D5, E6, E12, E18, FFC) was determined by the N-Phenyl-1-naphthylamine (NPN) assay. The 6 h grown *E. coli* was centrifuged (3500 rpm, 5 min) and resuspended at 4°C, washed and resuspended with 1 × PBS. This process was repeated twice. 150 µl *E. coli* suspension (−10^8^ CFU/ml) was then added to the wells of a 96-well black plate with clear bottom. Then 50 µl of 10 µM NPN was transferred to the wells and pre-incubated for 40 min. The fluorescence (350 nm/420 nm) was monitored every 2 min for 8 min. Then *E. coli* suspensions were added with 10 µl compound solution with E1, E2, E3, E4, E5, D5, E6, E12, E18, FFC and their final concentration were 6.25 µM. Fluorescence was detected immediately for another 12 min at every 2 min interval after adding compounds. The control group was the same as that of the experimental group except that the solvent without compound was added.

### Confocal Laser Scanning Microscopy

DAPI and PI dyes were used to evaluate the integrity of *E. coli* (ATCC 25922) membranes. *E. coli* (ATCC 25922) was adjusted to 1.0 × 107 CFU/ml with MHB during the exponential growth phase. The control group was not treated with bacteria. The bacteria were treated with samples at 4 × MIC and incubated for 2 h at 37°C. The bacterial suspension was then centrifuged (9000 g, 5 min). After removing the supernatant, the sediment was washed three times with 1×PBS. In the dark conditions, the bacteria were incubated with PI dyes (10 μg/ml) for 15 min at 4°C, and then the excess PI was washed twice with 1×PBS. The next step is to incubate the bacteria with DAPI (20 μg/ml) for 15 min at 4°C under dark conditions. Finally, the washed bacteria were suspended in 1×PBS. The suspensions were observed by confocal microscopy.

## Conclusion

Eighteen florfenicol-polyarginine conjugates were obtained by solid-phase and liquid-phase synthesis. Some of these conjugates demonstrated potent and broad antimicrobial activity. Florfenicol succinate showed certain activity after coupling with arginine, while florfenicol glutarate and adipate had no similar effect. Conjugating arginine to florfenicol succinate effectively modulated the properties of prodrugs. These conjugates were selectively toxic to bacterial cells compared with mammalian cells and had antibacterial activity in serum and plasma. The bacteriostatic effect of compound E6 was better than that of florfenicol in time-to-kill assays. These conjugates did not allow bacteria to develop resistance for bacterial growth inhibited by membrane depolarization and disruption. Therefore, these conjugates have a great promise to fight drug-resistance pathogens and provide useful enlightenment for rational designing novel antibiotics.

## Data Availability

The original contributions presented in the study are included in the article/Supplementary Material, further inquiries can be directed to the corresponding authors.
