# Supplementary material for: Florfenicol-Polyarginine Conjugates Exhibit Promising Antibacterial Activity Against Resistant Strains
Source: Front Chem. 2022 Jul 1;10:921091. doi: 10.3389/fchem.2022.921091 (PMC9284121; doi:10.3389/fchem.2022.921091)
Supplement: Supplementary file 1 [file DataSheet2.docx]

E1

| **Integration Peak List** | | |  |  |  |  |  |  |  |  |  |  |
| --- | --- | --- | --- | --- | --- | --- | --- | --- | --- | --- | --- | --- |
| **Peak** | **Start** | **RT** | **End** | **Height** | | | **Area** | | | **Area %** | | |
| 1 | 18.76 | 19.62 | 20.78 | 242.84 | | | 2880.35 | | | 100 | | |

E2

| **Integration Peak List** | |  |  |  |  |  |  |  |  |  |  |  |  |  |
| --- | --- | --- | --- | --- | --- | --- | --- | --- | --- | --- | --- | --- | --- | --- |
| **Peak** | **Start** | **RT** | **End** | | | **Height** | | | **Area** | | | **Area %** | | |
| 1 | 12.3 | 12.707 | 12.9 | | | 89.49 | | | 844.71 | | | 6.26 | | |
| 2 | 14.167 | 14.48 | 15.153 | | | 1289.77 | | | 13495.59 | | | 100 | | |

E3

| **Integration Peak List** | | |  |  |  |  |  |  |  |  |  |  |  |  |
| --- | --- | --- | --- | --- | --- | --- | --- | --- | --- | --- | --- | --- | --- | --- |
| **Peak** | **Start** | **RT** | **End** | | **Height** | | | **Area** | | | **Area %** | | |  |
| 1 | 9.473 | 10.14 | 10.887 | | 1239.07 | | | 8605.75 | | | 100 | | |  |

E4

| **Integration Peak List** | | | |  |  |  |  |  |  |  |  |  |  |  |  |  |
| --- | --- | --- | --- | --- | --- | --- | --- | --- | --- | --- | --- | --- | --- | --- | --- | --- |
| **Peak** | **Start** | **RT** | **End** | | | **Height** | | | **Area** | | | **Area %** | | |  |  |
| 1 | 8.66 | 9.56 | 10.073 | | | 1085.88 | | | 7493.72 | | | 100 | | |  |  |

E5

| **Integration Peak List** | | |  |  |  |  |  |  |  |  |  |  |  |  |  |  |
| --- | --- | --- | --- | --- | --- | --- | --- | --- | --- | --- | --- | --- | --- | --- | --- | --- |
| **Peak** | **Start** | **RT** | **End** | | **Height** | | | **Area** | | | **Area %** | | |  |  |  |
| 1 | 8.693 | 9.407 | 10.253 | | 1629.39 | | | 13126.54 | | | 100 | | |  |  |  |

E6

| **Integration Peak List** | | |  |  |  |  |  |  |  |  |  |  |  |  |  |  |
| --- | --- | --- | --- | --- | --- | --- | --- | --- | --- | --- | --- | --- | --- | --- | --- | --- |
| **Peak** | **Start** | **RT** | **End** | | **Height** | | | **Area** | | | **Area %** | | |  |  |  |
| 1 | 8.54 | 9.453 | 10.373 | | 1597.54 | | | 12453.52 | | | 100 | | |  |  |  |

E7

| **Integration Peak List** | | |  |  |  |  |  |  |  |  |  |  |  |  |  |
| --- | --- | --- | --- | --- | --- | --- | --- | --- | --- | --- | --- | --- | --- | --- | --- |
| **Peak** | **Start** | **RT** | **End** | | **Height** | | | **Area** | | | **Area %** | | |  |  |
| 1 | 10.62 | 11.387 | 12.12 | | 1538.17 | | | 10079.08 | | | 100 | | |  |  |

E8

| **Integration Peak List** | |  |  |  |  | |  |  |  | |  |  |  | |  |  |  | |  | |  | |
| --- | --- | --- | --- | --- | --- | --- | --- | --- | --- | --- | --- | --- | --- | --- | --- | --- | --- | --- | --- | --- | --- | --- |
| **Peak** | **Start** | **RT** | **End** | | | **Height** | | | | **Area** | | | | **Area %** | | | |  | |  | |  |
| 1 | 21.473 | 22.067 | 22.707 | | | 29.8 | | | | 81.23 | | | | 0.69 | | | |  | |  | |  |
| 2 | 24.453 | 24.88 | 25.747 | | | 886.98 | | | | 11757.95 | | | | 100 | | | |  | |  | |  |

E9

| **Integration Peak List** | | |  |  |  |  |  |  |  |  |  |  |  |  |
| --- | --- | --- | --- | --- | --- | --- | --- | --- | --- | --- | --- | --- | --- | --- |
| **Peak** | **Start** | **RT** | **End** | | **Height** | | | **Area** | | | **Area %** | | |  |
| 1 | 10.733 | 10.86 | 11.12 | | 991.35 | | | 5945.94 | | | 100 | | |  |

E10

| **Integration Peak List** | | |  |  |  |  |  |  |  |  |  |  |  |  |
| --- | --- | --- | --- | --- | --- | --- | --- | --- | --- | --- | --- | --- | --- | --- |
| **Peak** | **Start** | **RT** | **End** | **Height** | | | **Area** | | | **Area %** | | |  |  |
| 1 | 7.053 | 8.327 | 9.293 | 541.27 | | | 2570.42 | | | 100 | | |  |  |

E11

| **Integration Peak List** | | |  |  |  |  |  |  |  |  |  |  |  |  |  |
| --- | --- | --- | --- | --- | --- | --- | --- | --- | --- | --- | --- | --- | --- | --- | --- |
| **Peak** | **Start** | **RT** | **End** | | **Height** | | | **Area** | | | **Area %** | | |  |  |
| 1 | 9.867 | 10.467 | 11.52 | | 1557.1 | | | 11490.11 | | | 100 | | |  |  |

E12

| **Integration Peak List** | | | | | | |  |  |  |  |  |  |  |  |  |  |  |  |  |
| --- | --- | --- | --- | --- | --- | --- | --- | --- | --- | --- | --- | --- | --- | --- | --- | --- | --- | --- | --- |
| **Peak** | | **Start** | | **RT** | | **End** | | **Height** | | | **Area** | | | **Area %** | | |  |  |  |
| 1 | | 5.42 | | 5.533 | | 5.84 | | 1.65 | | | 6.45 | | | 0.14 | | |  |  |  |
| 2 | | 7.527 | | 8.133 | | 8.84 | | 912.25 | | | 4562.56 | | | 100 | | |  |  |  |
|  |  |  |  |  |  |  |  |  |  |  |  |  |  |  |  |  |  |  |  |

E13

| **Integration Peak List** | |  |  |  |  | |  |  |  | |  |  |  | |  |  |  | |  | |  | |
| --- | --- | --- | --- | --- | --- | --- | --- | --- | --- | --- | --- | --- | --- | --- | --- | --- | --- | --- | --- | --- | --- | --- |
| **Peak** | **Start** | **RT** | **End** | | | **Height** | | | | **Area** | | | | **Area %** | | | |  | |  | |  |
| 1 | 21.14 | 21.367 | 21.6 | | | 67.91 | | | | 666.13 | | | | 2.89 | | | |  | |  | |  |
| 2 | 21.813 | 21.993 | 22.753 | | | 1452.66 | | | | 23024.91 | | | | 100 | | | |  | |  | |  |

E14

| **Integration Peak List** | | |  |  |  |  |  |  |  |  |  |  |  |  |  |  |
| --- | --- | --- | --- | --- | --- | --- | --- | --- | --- | --- | --- | --- | --- | --- | --- | --- |
| **Peak** | **Start** | **RT** | **End** | | | **Height** | | | **Area** | | | **Area %** | | |  |  |
| 1 | 10.24 | 11.007 | 12.473 | | | 316.42 | | | 1230.27 | | | 100 | | |  |  |

E15

| **Integration Peak List** | | |  |  |  |  |  |  |  |  |  |  |  |  |  |  |
| --- | --- | --- | --- | --- | --- | --- | --- | --- | --- | --- | --- | --- | --- | --- | --- | --- |
| **Peak** | **Start** | **RT** | **End** | | | **Height** | | | **Area** | | | **Area %** | | |  |  |
| 1 | 10.04 | 11.127 | 11.727 | | | 1265.38 | | | 10018.68 | | | 100 | | |  |  |
| 2 | 12.24 | 12.52 | 12.807 | | | 12.28 | | | 71.11 | | | 0.71 | | |  |  |

E16

| **Integration Peak List** | |  |  |  |  | |  |  |  | |  |  |  | |  |  |  | |  | |  | |
| --- | --- | --- | --- | --- | --- | --- | --- | --- | --- | --- | --- | --- | --- | --- | --- | --- | --- | --- | --- | --- | --- | --- |
| **Peak** | **Start** | **RT** | **End** | | | **Height** | | | | **Area** | | | | **Area %** | | | |  | |  | |  |
| 1 | 10.013 | 10.46 | 11.247 | | | 819.9 | | | | 8861.81 | | | | 100 | | | |  | |  | |  |
| 2 | 12.24 | 12.487 | 12.84 | | | 76.74 | | | | 468.22 | | | | 5.28 | | | |  | |  | |  |

E17

| **Integration Peak List** | | |  |  |  |  |  |  |  |  |  |  |  |  |  |
| --- | --- | --- | --- | --- | --- | --- | --- | --- | --- | --- | --- | --- | --- | --- | --- |
| **Peak** | **Start** | **RT** | **End** | | | **Height** | | | **Area** | | | **Area %** | | |  |
| 1 | 8.66 | 10.267 | 11.307 | | | 996.12 | | | 10668.71 | | | 100 | | |  |

E18

| **Integration Peak List** | | |  |  |  |  |  |  |  |  |  |  |  |  |  |  |
| --- | --- | --- | --- | --- | --- | --- | --- | --- | --- | --- | --- | --- | --- | --- | --- | --- |
| **Peak** | **Start** | **RT** | **End** | | | **Height** | | | **Area** | | | **Area %** | | |  |  |
| 1 | 9.687 | 10.233 | 10.947 | | | 1148.57 | | | 12144.61 | | | 100 | | |  |  |
