# Supplementary material for: Florfenicol-Polyarginine Conjugates Exhibit Promising Antibacterial Activity Against Resistant Strains
Source: Front Chem. 2022 Jul 1;10:921091. doi: 10.3389/fchem.2022.921091 (PMC9284121; doi:10.3389/fchem.2022.921091)

^1^H NMR, ^1^C NMR and LC-MS spectra for all compounds

Compound C1(^1^H NMR)

Compound C1 (^1^C NMR)

C2 (^1^H NMR)

C2 (^1^C NMR)

C3 (^1^H NMR)

C3 (^1^C NMR)

D3 (^1^H NMR)

D3 (^1^C NMR)

D4 (^1^H NMR)

D4 (^1^C NMR)

D5 (^1^H NMR)

D5 (^1^C NMR)

D6 (^1^H NMR)

D6 (^1^C NMR)

E1 (^1^H NMR)

E1 (^1^C NMR)


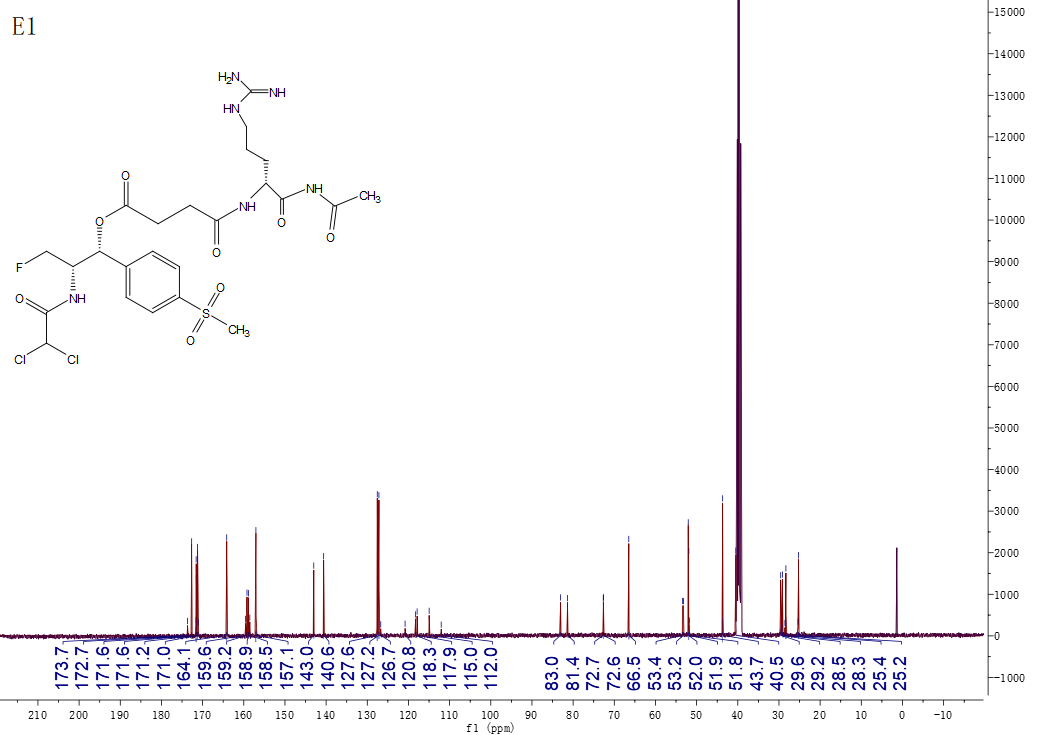


E2 (^1^H NMR)

E2 (^1^C NMR)

E3 (^1^H NMR)

E3 (^1^C NMR)

E4 (^1^H NMR)

E4 (^1^C NMR)

E5 (^1^H NMR)

E5 (^1^C NMR)

E6 (^1^H NMR)

E6 (^1^C NMR)

E7 (^1^H NMR)

E7 (^1^C NMR)

E8 (^1^H NMR)

E8 (^1^C NMR)

E9 (^1^H NMR)

E9 (^1^C NMR)

E10 (^1^H NMR)

E10 (^1^C NMR)

E11 (^1^H NMR)

E11 (^1^C NMR)

E12 (^1^H NMR)

E12 (^1^C NMR)

E13 (^1^H NMR)

E13 (^1^C NMR)

E14 (^1^H NMR)

E14 (^1^C NMR)

E15 (^1^H NMR)

E15 (^1^C NMR)

E16 (^1^H NMR)

E16 (^1^C NMR)

E17 (^1^H NMR)

E17 (^1^C NMR)

E18 (^1^H NMR)

E18 (^1^C NMR)

LC-MS spectra

C1


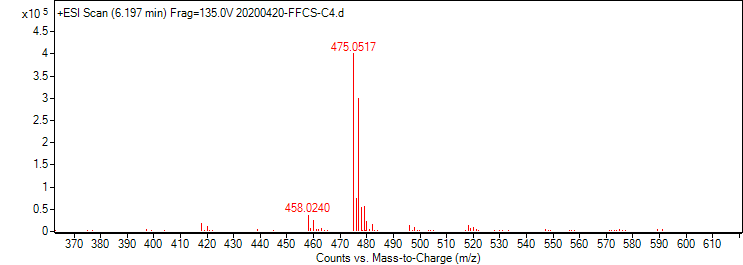


C2


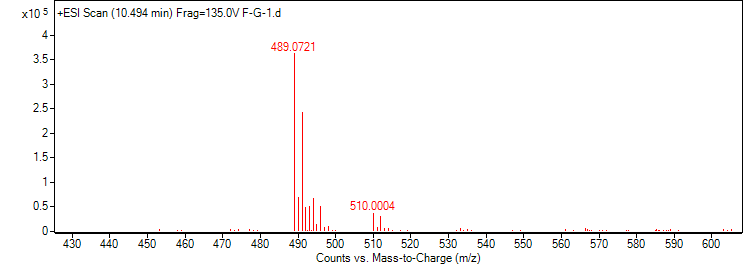
C3


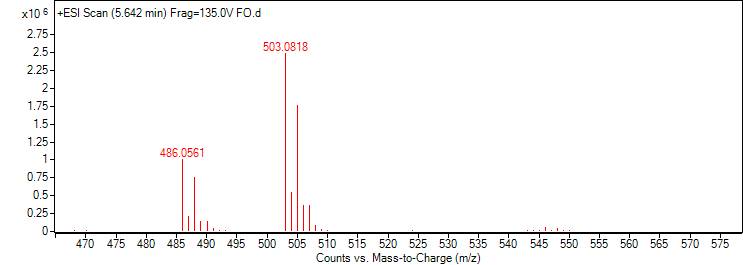
D1
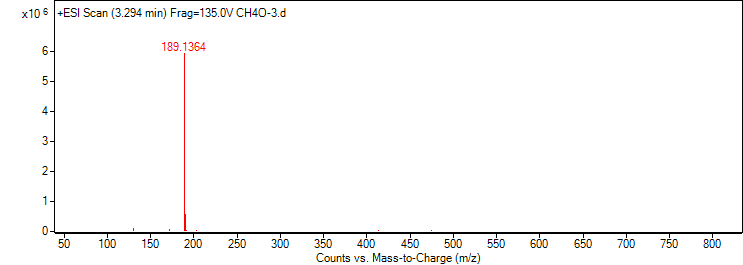


D2


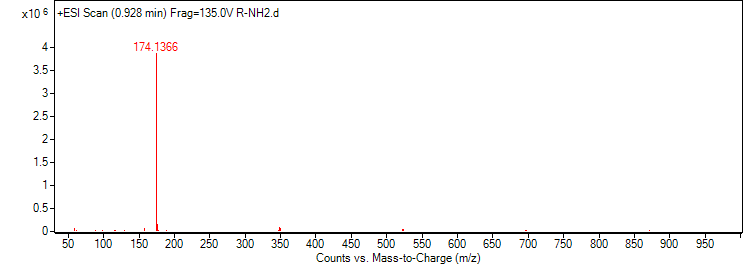


D3 (165.6208; 2H+)


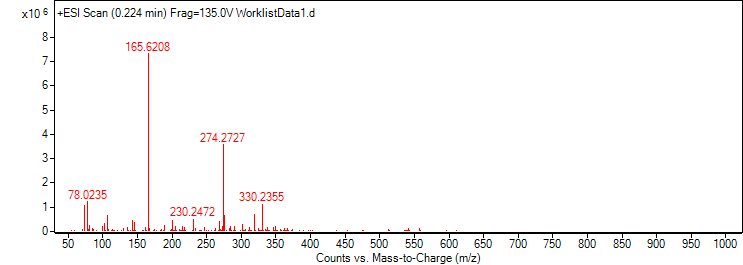


D4 （161.3646; 4H+）


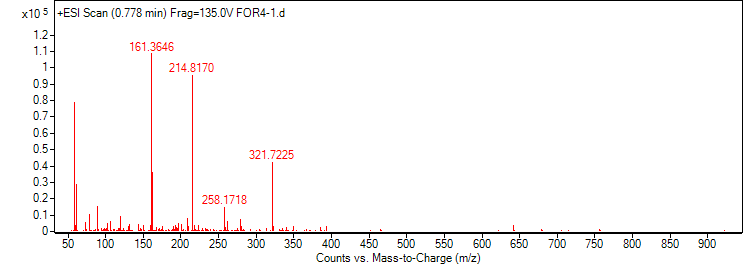


D5 (239.4157; 4H+)


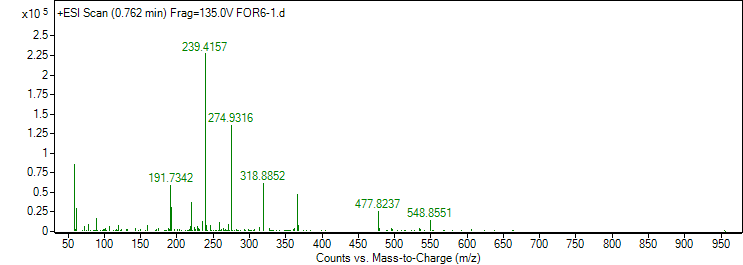


D6 (254.1753; 5H+)


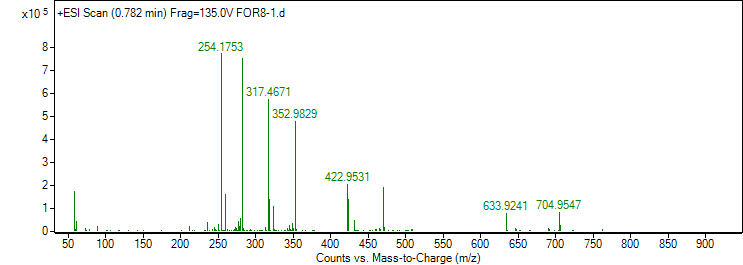


E1


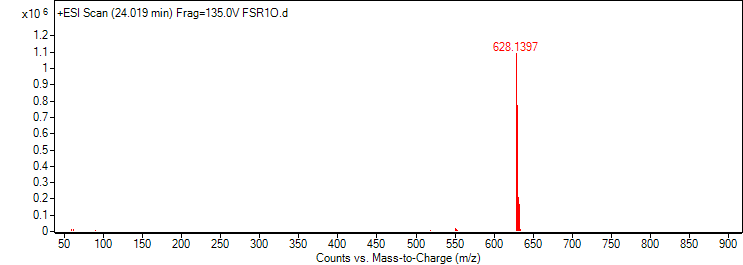


E2


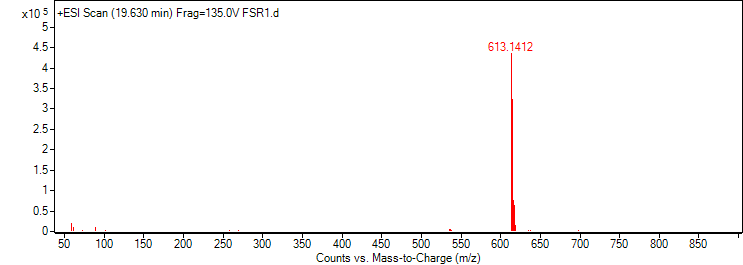


E3


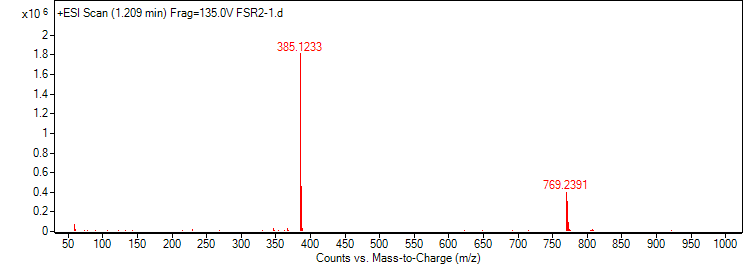


E4


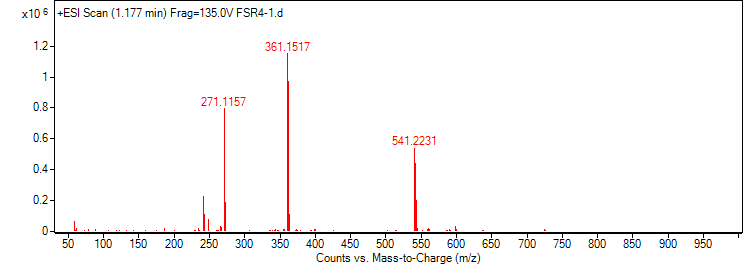


E5


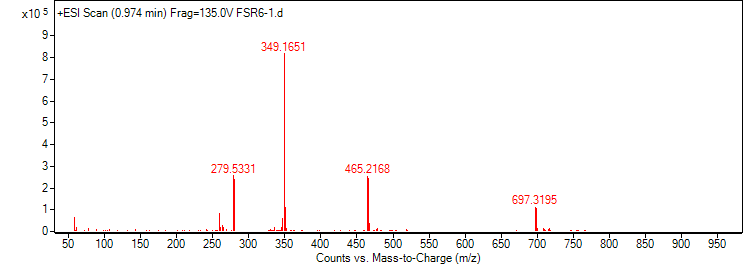


E6


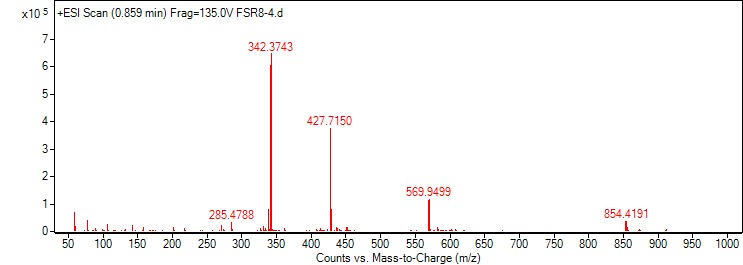


E7


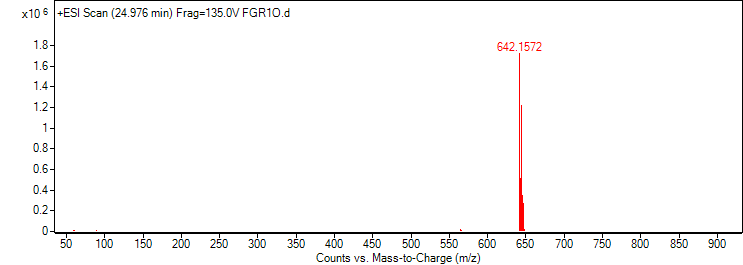


E8


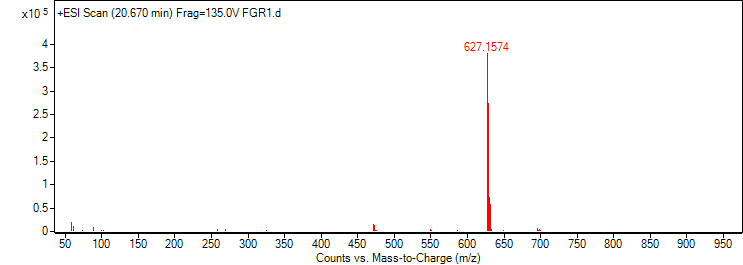


E9


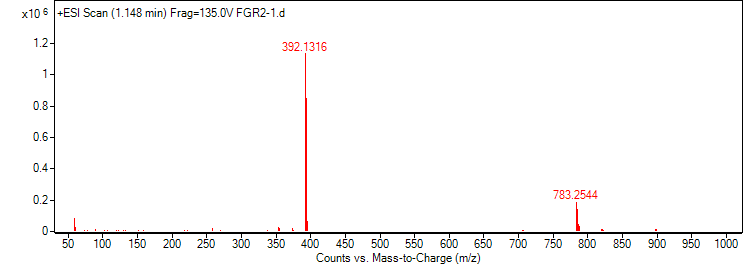


E10


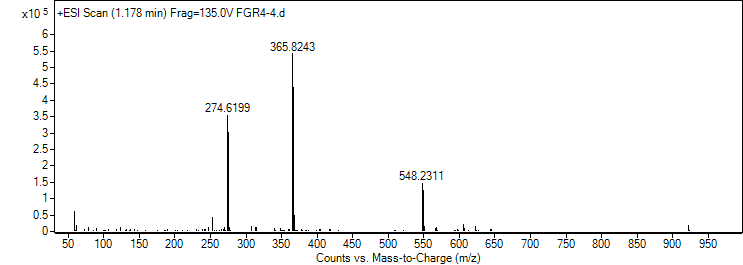


E11


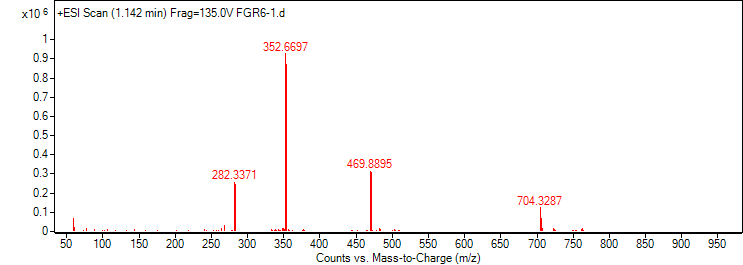


E12


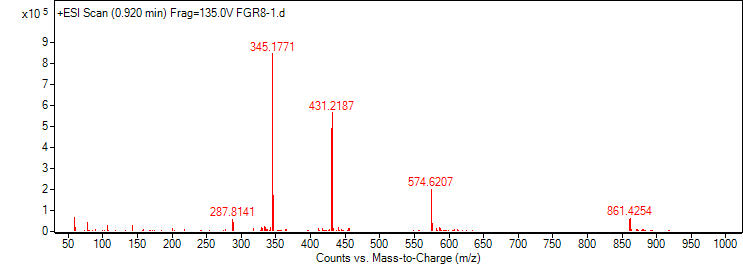


E13


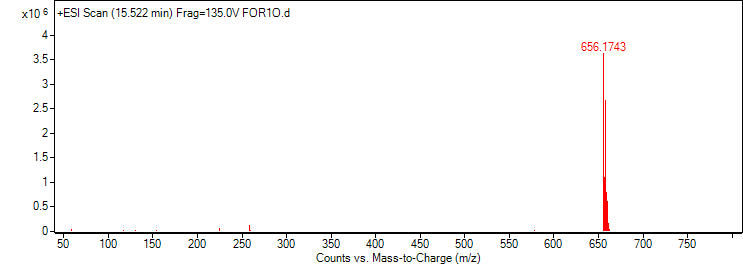
E14


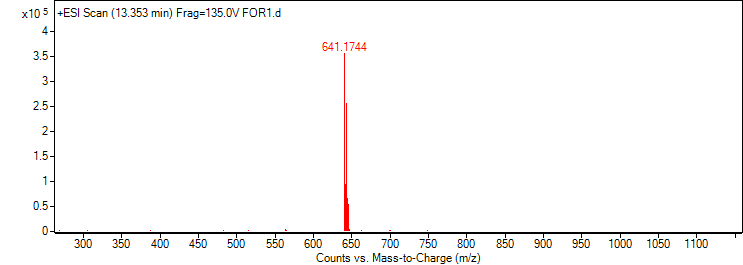
E15


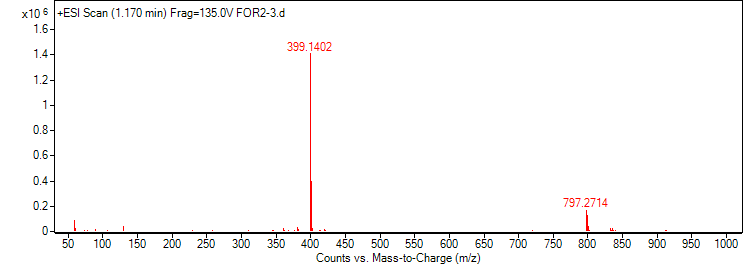


E16


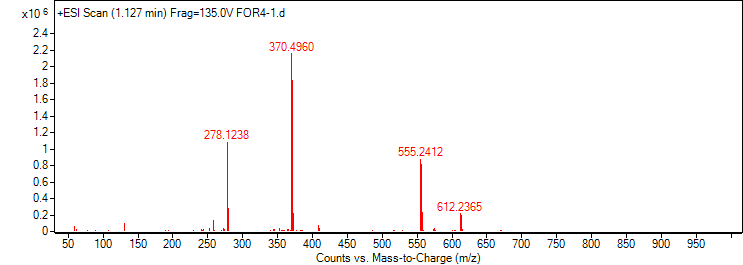


E17


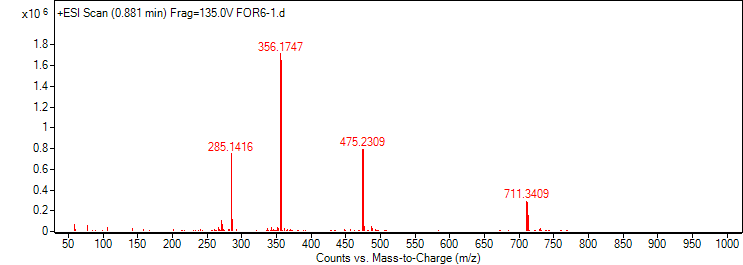


E18
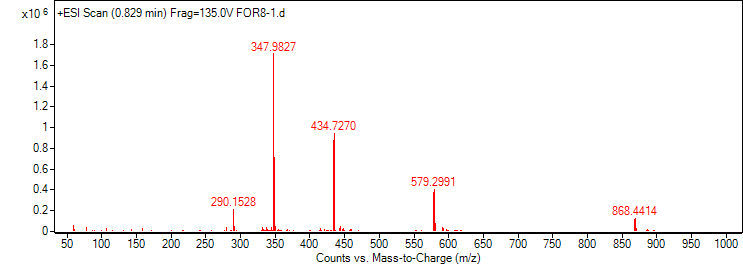

Supplement: Supplementary file 2 [file DataSheet1.docx]
